# Intermediate Transfer Rates and Solid-State Ion Exchange are Key Factors Determining the Bifunctionality of In_2_O_3_/HZSM-5 Tandem CO_2_ Hydrogenation Catalyst

**DOI:** 10.1021/acssuschemeng.3c08250

**Published:** 2024-03-18

**Authors:** Fatima Mahnaz, Jasan Robey Mangalindan, Balaji C. Dharmalingam, Jenna Vito, Yu-Ting Lin, Mustafa Akbulut, Jithin John Varghese, Manish Shetty

**Affiliations:** † Artie McFerrin Department of Chemical Engineering, Texas A&M University, 100 Spence Street, College Station, Texas 77843, United States; ‡ Department of Chemical Engineering, Indian Institute of Technology Madras, Chennai, Tamil Nadu 600036, India

**Keywords:** CO_2_ utilization, sustainable fuels, C−C coupling, zeolite, ion-exchange, methanol, hydrocarbon

## Abstract

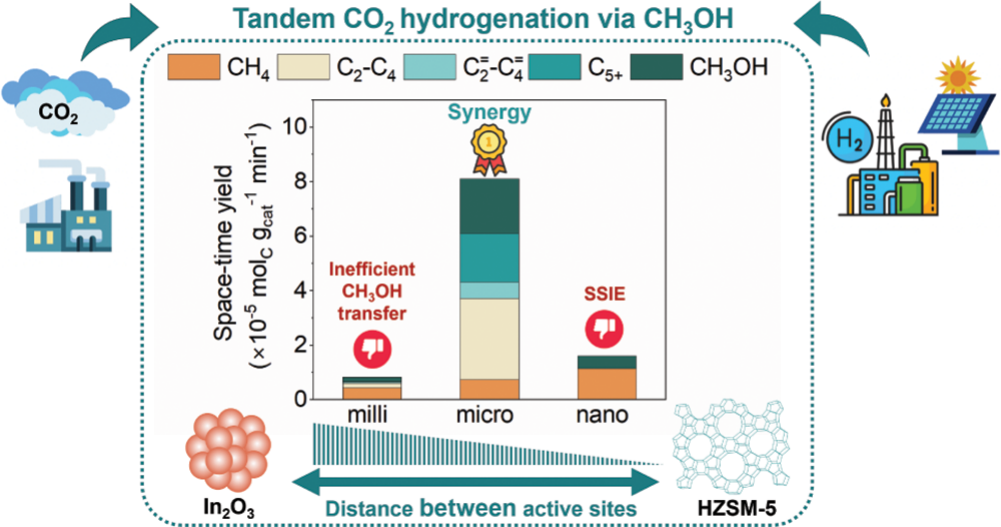

Identifying the descriptors for the synergistic catalytic activity of bifunctional oxide-zeolite catalysts constitutes a formidable challenge in realizing the potential of tandem hydrogenation of CO_2_ to hydrocarbons (HC) for sustainable fuel production. Herein, we combined CH_3_OH synthesis from CO_2_ and H_2_ on In_2_O_3_ and methanol-to-hydrocarbons (MTH) conversion on HZSM-5 and discerned the descriptors by leveraging the distance-dependent reactivity of bifunctional In_2_O_3_ and HZSM-5 admixtures. We modulated the distance between redox sites of In_2_O_3_ and acid sites of HZSM-5 from milliscale (∼10 mm) to microscale (∼300 μm) and observed a 3-fold increase in space-time yield of HC and CH_3_OH (7.5 × 10^–5^ mol_C_ g_cat_^–1^ min^–1^ and 2.5 × 10^–5^ mol_C_ g_cat_^–1^ min^–1^, respectively), due to a 10-fold increased rate of CH_3_OH advection (1.43 and 0.143 s^–1^ at microscale and milliscale, respectively) from redox to acid sites. Intriguingly, despite the potential of a three-order-of-magnitude enhanced CH_3_OH transfer at a nanoscale distance (∼300 nm), the sole product formed was CH_4_. Our reactivity data combined with Raman, Fourier transform infrared (FTIR), and X-ray photoelectron spectroscopy (XPS) revealed the occurrence of solid-state-ion-exchange (SSIE) between acid sites and In^δ+^ ions, likely forming In_2_O moieties, inhibiting C–C coupling and promoting CH_4_ formation through CH_3_OH hydrodeoxygenation (HDO). Density functional theory (DFT) calculations further revealed that CH_3_OH adsorption on the In_2_O moiety with preadsorbed and dissociated H_2_ forming an H–In–OH–In moiety is the likely reaction mechanism, with the kinetically relevant step appearing to be the hydrogenation of the methyl species. Overall, our study revealed that efficient CH_3_OH transfer and prevention of ion exchange are the key descriptors in achieving catalytic synergy in bifunctional In_2_O_3_/HZSM-5 systems.

## Abstract 2

Efficient CH_3_OH transfer and prevention of ion-exchange between zeolitic H^+^ and In^δ+^ drive catalytic synergy over In_2_O_3_/HZSM-5 bifunctional admixtures.

## Introduction

In 2023, NASA’s Goddard Institute of Space Studies (GISS) in New York reported that the summer marked Earth’s highest temperatures on record since 1880.^1^ This warming trend was predominantly driven by anthropogenic CO_2_ emissions.^1^ As per the United Nations, the atmospheric CO_2_ level has seen a 50% increase over preindustrial levels, reaching 421 ppm in May 2022.^2−4^ A potential approach for decreasing CO_2_ levels in the atmosphere is the conversion of CO_2_ into value-added hydrocarbons (HC), fuel, and chemicals by tandem catalysis.^5−8^

While the idea of converting small molecules into larger and more complex ones seems appealing, it often requires reactions occurring over different active sites (e.g., acylation of aldehydes on Lewis acid and base sites, hydrogenation–hydroformylation over central nitrogen in tertiary amine on metal sites, and alkene metathesis on redox and acid sites).^9−20^ Even so, tandem (cascade or domino) reactions have proven effective in facilitating such complex transformations in a single step by utilizing multifunctional catalysts.^21−23^ Examples of such reactions include tandem dehydrogenation and olefin cross-metathesis,^24−28^ tandem ammonia borane dehydrogenation and hydrogenation of amines,^29^ and tandem hydrogenation of nitroarenes.^30^ Tandem reactions are further attractive as they promote process intensification, reducing capital and operational costs by consolidating multiple reaction steps in a single reactor.^31−37^ As such, tandem hydrogenation of carbon dioxide (CO_2_) with “green H_2_”, which couples two major reactions: CH_3_OH synthesis reaction and CH_3_OH to hydrocarbons conversion (MTH), to produce HC has emerged as an attractive route to advance toward a sustainable and carbon-neutral circular economy.^7,38−52^

The design of efficient catalysts for the selective hydrogenation of CO_2_ faces two major challenges, (i) the constituent steps within the tandem reaction require different catalytic active sites, such as acid and redox sites, which are unlikely to be found within a single material^53−56^ and (ii) a specific reaction sequence needs to be followed to achieve the desired product (e.g., CH_3_OH synthesis has to occur before MTH).^57−60^ The former can be addressed by incorporating the necessary active sites in a single material (i.e., bifunctional catalysts). However, designing efficient tandem catalysts requires careful consideration of factors beyond the inclusion of active sites. Specifically, the second constraint suggests that the mere inclusion of necessary active sites in the catalyst is inadequate as efficient conversion requires the transport of reactants and intermediates from and to specific active sites in a specific reaction sequence. This is apparent considering most of the “state-of-the-art” bifunctional catalysts reported for tandem CO_2_ hydrogenation are simple admixtures, which address the requirement of multiple active sites, e.g., oxygen vacancies (redox sites) on a metal oxide and Bro̷nsted acid sites (BAS) on a zeolite yet exhibit poor control over the spatial arrangements of active sites to synchronize the sequence of reaction steps. However, simple bifunctional admixture catalysts can still exhibit increased selectivity toward hydrocarbon products,^5,61−66^ making it imperative to decipher the factors that drive catalytic synergy in these admixtures to determine the optimal catalyst heterostructures.

The key challenge in probing the synergy involved in tandem CO_2_ hydrogenation is the complexity of the overall reaction network.^67^ While the first step (CH_3_OH synthesis) causes a few side reactions of reverse water gas shift (RWGS) and CO_2_ methanation,^68,69^ the second step (MTH) encompasses a series of reactions such as the formation of C–C bond, formation of lower olefin, olefin methylation, olefin cracking, hydrogen transfer, cyclization, aromatic methylation, aromatic dealkylation, formation of alkanes by secondary hydrogenation, and co-catalytic intermediates formation in the olefin and aromatic cycles of the “dual-cycle mechanism”.^70^ This complex reaction system poses challenges in identifying the specific steps that can aid in achieving synergistic performance to enhance the selectivity of specific hydrocarbons.

Probing the catalytic synergy is further complex as the proximity and compatibility between the redox sites and BAS in the admixtures are vital for the efficient hydrogenation of CO_2_. There is a consensus that the HC selectivity could be enhanced by improving the transfer of CH_3_OH intermediate from redox sites to BAS by reducing the spatial distances between them; in other words, “the closer, the better”.^39,71,72^ These improvements have only been shown as improved HC selectivity.^39,71−75^ The influence of the increased CH_3_OH advection rates on the HC production rate (i.e., HC space-time yields) has not been enumerated yet. Additionally, several studies have shown that the intimate proximity between the active sites was detrimental to CO_2_ hydrogenation, especially for In_2_O_3_ and zeolite admixtures.^7,74,76−78^ This change in catalytic activity was suggested to be caused by multiple factors, including the destruction of zeolite structure,^77^ the reduction of metal oxides (e.g., In_2_O_3_) by hydrogen during CO_2_ hydrogenation,^40^ and/or cation (e.g., In^δ+^) migration and exchange with BAS under harsh reaction conditions.^76,79^ Recently, a few reports have shown the effect of ion exchange on the inhibition of the catalytic rate of HC production.^7,74,80^ However, systematic investigation of ion-exchange, formation of cationic species inside the zeolite, and their influence on HC yields and the reaction mechanism are yet to be carried out.

Considering all the factors required in designing an effective catalyst, in this study, we seek to establish (1) a comprehensive understanding of how the placement of the active sites manipulates the reaction trajectories in the complex reaction network of CH_3_OH and HC formation and influences HC space-time yields (2) a quantitative analysis of how the rate of CH_3_OH advection from redox to BAS influences the yields and selectivity of HC; (3) at what conditions ion exchange may occur and how different extents of ion exchange can influence the product selectivity and yields and (4) if ion exchange can create new active sites in the zeolite framework and promote side reactions during tandem CO_2_ hydrogenation. We selected In_2_O_3_ and HZSM-5 (Si/Al = 40) for their efficacy in CH_3_OH synthesis,^81^ and MTH conversion,^82,83^ respectively, and modulated the placement of redox and BAS in the admixtures (from milliscale to nanoscale) to evaluate the effect of their proximity on the reaction pathways and product yield and selectivity. Overall, we aimed to explore the factors that determine the catalytic synergy in bifunctional admixtures during tandem CO_2_ hydrogenation.

## Experimental Section

### Materials

Indium(III) nitrate hydrate (99.999% metal basis, Thermo Scientific chemicals, Richardson, Texas, USA) and ammonium hydroxide (28–30% NH_3_ basis, Sigma-Aldrich, St. Louis, Missouri, USA) were used to synthesize indium oxide (In_2_O_3_). Zeolite Socony Mobil–5 (NH_4_-ZSM-5, CBV 8014, Si:Al ratio 40) zeolite was purchased from Zeolyst (Kansas City, USA). Sodium nitrate (ReagentPlus, ≥ 99%, Sigma-Aldrich, St. Louis, Missouri, USA) was used for ion exchange with HZSM-5. Fused α-Alumina (100–200 mesh, Sigma-Aldrich, St. Louis, Missouri, US) was used for spacing in stacked bed catalysts.

### Catalyst Synthesis

#### Synthesis of Indium Oxide (In_2_O_3_)

Indium oxide (In_2_O_3_) was synthesized by the precipitation method.^74,76^ Briefly, indium(III) nitrate trihydrate (In(NO_3_)_3_.3H_2_O, 5 g) was added to deionized (DI) water (20 mL). The solution was added dropwise to an ammonium hydroxide (NH_4_OH) solution (60 mL, 0.8 M). The as-prepared mixture was aged overnight (70 °C, 12 h). The mixture was then filtered under a vacuum. The filtrate/precipitate was then washed with ethanol (70%), dried (80 °C, 5 h), and calcined (500 °C, 4 h) with air (50 mL min^–1^) in a muffle furnace.

#### Preparation of HZSM-5

NH_4_-ZSM-5 was calcined (500 °C, 4 h) under air (50 mL min^–1^) in a muffle furnace to produce HZSM-5. Specifically, the temperature was increased from 20 to 80 °C (ramp rate of 10 °C/min), followed by an isothermal holdup at 80 °C for 6 h for drying, and then ramped up to 500 °C with a ramp of 19 °C/min, followed by an isothermal holdup at 500 °C for 4 h.

#### Ion-Exchange of HZSM-5 with Indium (*x*InZSM-5)

Three ion-exchanged *x*InZSM-5 samples with different In:Al molar ratios (*x*= 0.3, 0.7, 3.5) were prepared by incipient wetness impregnation (IWI) of freshly calcined HZSM-5 (3.5 g) with a solution of In(NO_3_)_3_.3H_2_O (0.03, 0.08, and 1.5 g in 1 mL H_2_O for In:Al ratio of 0.3, 0.7, and 3.5, respectively). The mixture was then dried (80 °C, 5 h) and calcined (500 °C, 6 h) with air (50 mL min^–1^) in a muffle furnace.

#### Ion-Exchange of HZSM-5 with Sodium (NaZSM-5)

HZSM-5 was ion-exchanged with Na^+^ using the wetness impregnation method.^84^ Specifically, a 2 M NaNO_3_ solution was prepared by adding 6.8 g NaNO_3_ in 40 g of DI water. Two grams of HZSM-5 were added to the solution, and the mixture was stirred for 2 h at 70 °C. The mixture was filtered and washed with DI water. The zeolite was collected, and the ion exchange procedure was repeated 4 times. The final ion-exchanged zeolite was then washed, dried (80 °C, 5 h), and calcined (500 °C, 6 h) with air (50 mL min^–1^) in a muffle furnace.

### Preparation of Bifunctional In_2_O_3_/HZSM-5 Admixtures

#### Microscale Placement of In_2_O_3_ and HZSM-5 (micro_In_2_O_3_/HZSM-5)

The admixture was prepared by physically mixing granules of In_2_O_3_ and HZSM-5 at a mass ratio of 1:1 (1 g total, unless otherwise specified). To prepare the granules, the powder of In_2_O_3_ and HZSM-5 were separately pressed, crushed, and sieved into 30–60 mesh (size 250–560 μm).

#### Nanoscale Placement of In_2_O_3_ and HZSM-5 (nano_In_2_O_3_/HZSM-5)

The nanoscale admixture was prepared by mixing In_2_O_3_ and HZSM-5 powder (1 g total, 1:1 mass ratio) in an agate mortar and pestle for 15 min, followed by pressing, crushing, and sieving into granules of 30–60 mesh (size 250–560 μm).

#### Stacked Bed of In_2_O_3_ and HZSM-5 (In_2_O_3_||HZSM-5 and HZSM-5||In_2_O_3_)

In_2_O_3_ and H-ZSM-5 (0.5 g each) were separately pressed, crushed, and sieved into granules of 30–60 meshes (250–560 μm). Then, the In_2_O_3_ and HZSM-5 granules were stacked as separate beds with fused α-alumina (Al_2_O_3_) (0.5 g) in between.

### Catalytic Evaluation Methods

The catalytic conversion of CO_2_ hydrogenation was evaluated on a high-pressure tubular fixed-bed reactor. Typically, the catalyst (1.0 g, 30–60 mesh) was first pretreated in 5% H_2_ (balance N_2_) at 300 °C for 1 h and cooled to 40 °C prior to the reaction. We note that blank reaction tests with α-Al_2_O_3_ showed no reactivity. The reaction was conducted at a pressure of 500 psig and a temperature of 350 °C, unless otherwise specified. Gas hourly space velocity (GHSV) was calculated using the following equation:
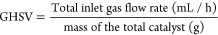
1

GHSV was maintained at 9000 mL g_cat_^–1^ h^–1^ with a 3:1 feed ratio of H_2_:CO_2_, unless otherwise specified.

The products were analyzed by an online gas chromatograph (SRI-GC Multigas 5) equipped with a flame ionization detector (FID), a methanizer (FIDm), and a thermal conductivity detector (TCD). A Haysep D column was connected to the TCD and FIDm for separating and detecting CO_2_, CO, CH_4_, and C_2_–C_4_ HC while the MXT-1 column was connected to the FID for analyzing all HC and oxygenate products (e.g., dimethyl ether and CH_3_OH). The outlet from the reactor was further analyzed by Agilent GCMS (8890 GC system and 5977B GC/MSD) equipped with a GasPro column connected to FID and mass spectrometer for further quantification and identification of the products. The product selectivity was calculated on a molar carbon basis. The carbon balance is given in the SI (Table S7).

The CO_2_ conversion, CO selectivity, HC distribution, and STY of HC were calculated using the following equations:
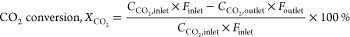
2
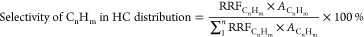
3
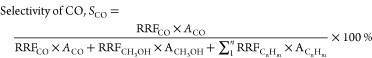
4
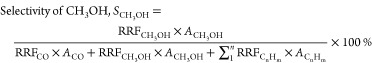
5
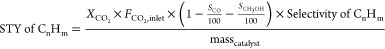
6where *C*_CO_2_, inlet_ and *C*_CO_2_, outlet_ are the concentrations of CO_2_ at the inlet and outlet, respectively, *F*_inlet_ and *F*_outlet_ are the inlet and outlet gas flow rates of the reactor, RRF is the relative response factor, *A* is the peak area of the species on the chromatogram, and *n* and *m* denote the number of C and H atoms in the HC.

For the catalytic performance evaluation in bar plots, the data were averaged over three points under a specific reaction condition. The carbon balance was done on a carbon mole basis. The carbon balance ranges from 96 to 101% as shown in SI Table S7.

MTH reaction was conducted in the same tubular fixed-bed reactor. The reaction was conducted at a pressure of 200 psi and a reaction temperature of 350 °C. GHSV was 9000 mL g_cat_^–1^ h^–1^ with a 3:1 feed ratio of H_2_:N_2_, keeping p_H2_ the same as in CO_2_ hydrogenation. CH_3_OH was injected using a high-pressure syringe pump (Chemyx Fusion 6000X) with a flow rate of 0.012 mL min^–1^.

## Results and Discussion

### Sequential Placement of Active Sites in Bifunctional In_2_O_3_ and HZSM-5 Admixtures

**Figure 1:**
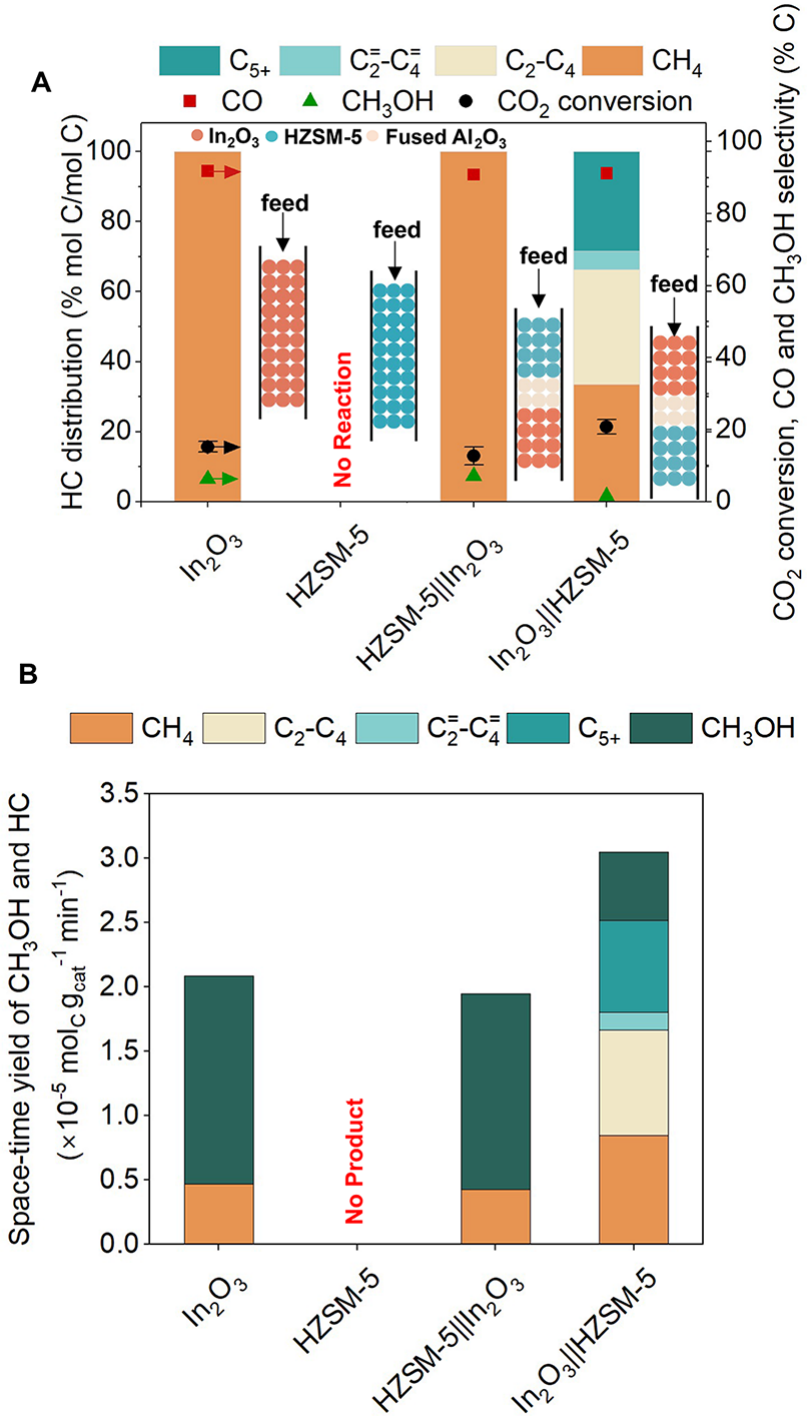
Requirement in the sequential placement of active sites in bifunctional In_2_O_3_and HZSM-5 admixtures. (A) Catalytic performance including hydrocarbon distribution (left axis), CO_2_ conversion, CO and CH_3_OH selectivity (right axis) during CO_2_ hydrogenation over In_2_O_3_, HZSM-5, HZSM-5||In_2_O_3_, and In_2_O_3_||HZSM-5 (inserts show configurations of the catalyst beds). (B) Space–time yield (STY) of CH_3_OH and HC over In_2_O_3_, HZSM-5, HZSM-5||In_2_O_3_, and In_2_O_3_||HZSM-5. Reaction conditions: 350 °C, 500 psig, 9000 mL g_cat_^–1^ h^–1^, H_2_:CO_2_ ratio 3:1, In_2_O_3_ and HZSM-5 mass ratio 1:1.

We assessed the catalytic performances of In_2_O_3_ and HZSM-5 for CO_2_ hydrogenation at 350 °C, 500 psig, and GHSV of 9000 mL g_cat_^–1^ h^–1^ (Figure 1A). In_2_O_3_ generated only C_1_ products, including CH_3_OH (6.3%), CO (91.8%), and CH_4_ (1.9%). The formation of CO and CH_4_ occurs via the RWGS and CO_2_ methanation, respectively.^81,85^ Due to the absence of BAS, no C_2+_ HC was formed over In_2_O_3_. On the other hand, HZSM-5 did not show any reactivity, which is consistent with previous reports, as the BAS of HZSM-5 alone does not catalyze CO_2_ hydrogenation.^39^

We next assessed the requirements of bifunctionality and sequential placement of active sites by evaluating the catalytic performance of stacked granules (∼ 405 μm) of In_2_O_3_ and HZSM-5 with a 3 mm inert layer of fused α-Al_2_O_3_ separating each catalyst in two arrangements, (i) In_2_O_3_ stacked on top of HZSM-5, denoted as In_2_O_3_||HZSM-5, and (ii) HZSM-5 stacked on top of In_2_O_3_, denoted as HZSM-5||In_2_O_3_ (see insets in Figure 1A). For HZSM-5||In_2_O_3_, akin to In_2_O_3,_ only C_1_ products were observed (91% CO, 7.1% CH_3_OH, and 1.9% CH_4_ in total product selectivity) as In_2_O_3_ was solely responsible for CO_2_ hydrogenation. However, we observed the formation of C_2+_ HC over In_2_O_3_||HZSM-5 (Figure 1A) with an HC distribution of ∼32.6% paraffins (C_2_–C_4_), ∼5.4% lower olefins (C_2_^=^–C_4_^=^), and ∼28.3% longer-chain HCs (C_5+_) which substantiates the requirement of bifunctionality for the tandem reaction. Taken together, our data suggests that the sequence of active sites in bifunctional catalysts is crucial for the efficient conversion of CO_2_ to HC.

The space–time yields (STY) of HC and CH_3_OH over In_2_O_3,_ In_2_O_3_||HZSM-5, and HZSM-5||In_2_O_3_ at 350 °C are shown in Figure 1B. The highest STY was observed over In_2_O_3_||HZSM-5 (3.00 × 10^–5^ mol_C_ g_cat_^–1^ min^–1^), compared to In_2_O_3_ (2.00 × 10^–5^ mol_C_ g_cat_^–1^ min^–1^) and HZSM-5||In_2_O_3_ (1.95 × 10^–5^ mol_C_ g_cat_^–1^ min^–1^), suggesting that the BAS of HZSM-5 converted the CH_3_OH formed over In_2_O_3_ (equilibrium limited under reaction conditions,^86^ see Section S4.2) via MTH, driving the overall reaction forward (as per Le Chatelier’s principle).^36,87^

### Influence of Active Site Proximity and Intermediate Transfer Rates on Catalytic Activity

We tuned the distance between the active sites of In_2_O_3_ and HZSM-5 (In_2_O_3_||HZSM-5, micro_In_2_O_3_/HZSM-5, and nano_In_2_O_3_/HZSM-5) to investigate the influence of their proximity on catalytic activity. The estimated average distances between redox sites and BAS in In_2_O_3_||HZSM-5, micro_In_2_O_3_/HZSM-5, and nano_In_2_O_3_/HZSM-5 (described in Section S2) were ∼9.6 mm, ∼318 μm, and ∼315 nm, respectively.

**Figure 2:**
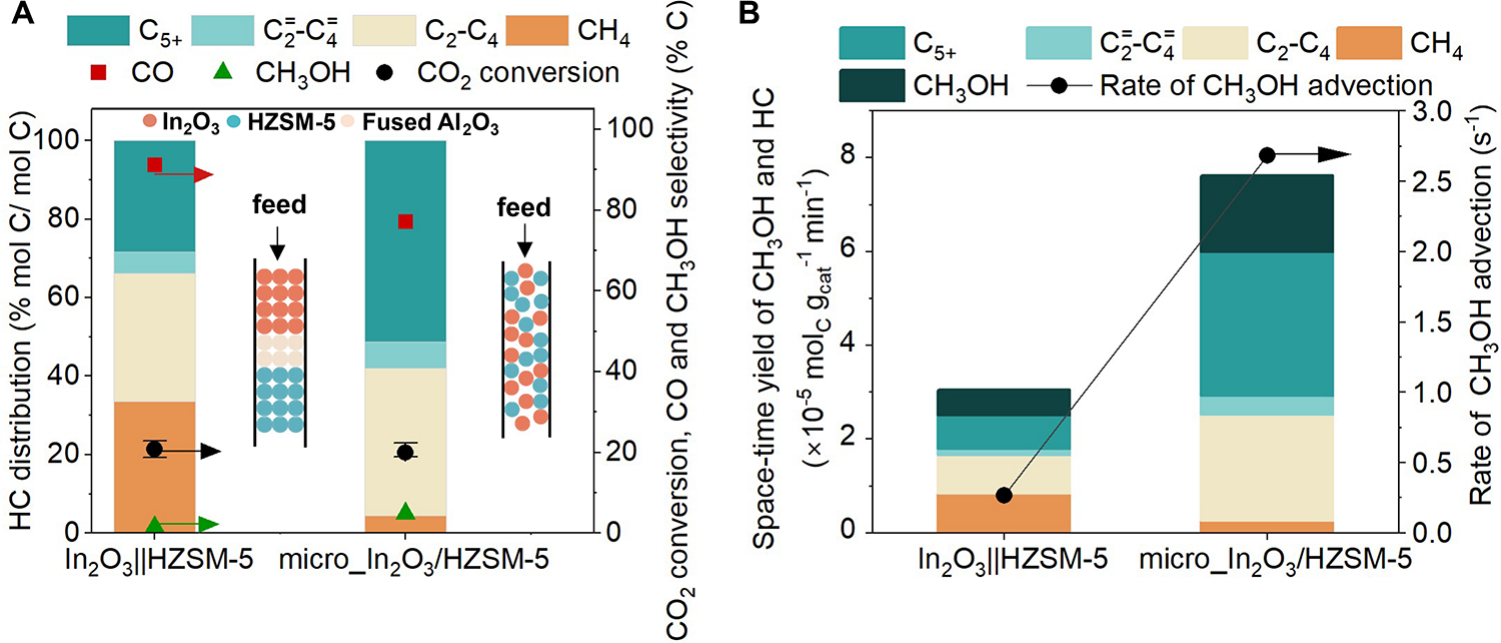
Influence of the proximity between redox sites of In_2_O_3_and Bro̷nsted acid sites (BAS) of HZSM-5 on catalytic activity. (A) Catalytic performance including hydrocarbon (HC) distribution (left axis), CO_2_ conversion, CO and CH_3_OH selectivity (right axis) during CO_2_ hydrogenation over In_2_O_3_||HZSM-5 and micro_In_2_O_3_/HZSM-5. (B) Space–time yield (STY) of CH_3_OH and HC (left axis) and rate of CH_3_OH advection (right axis) over In_2_O_3_||HZSM-5 and micro_In_2_O_3_/HZSM-5. Reaction conditions: 350 °C, 500 psig, 9000 mL g_cat_^–1^ h^–1^, H_2_:CO_2_ ratio 3:1, In_2_O_3_:HZSM-5 mass ratio 1:1.

Micro_In_2_O_3_/HZSM-5 exhibited decreased CO selectivity (77%) and a higher C_5+_ selectivity (51% in HC distribution) as compared to In_2_O_3_||HZSM-5 (CO selectivity of 91.0% and C_5+_ selectivity of 28.3%) at a similar CO_2_ conversion (20.0%) (see Figure 2A). We observed only branched alkanes and cyclic hydrocarbons and no aromatics in C_5+_ hydrocarbons over micro_In_2_O_3_/HZSM-5 (see Figure S22) likely due to the presence of high-pressure H_2_ during the reaction, which tends to hydrogenate olefins and aromatics over the BAS of the zeolite.^88^ This observation is consistent with previous reports for HZSM-5 (with Si:Al ratio 15 to 50) under similar reaction conditions.^76,89,90^ Interestingly, the combined STY of CH_3_OH and HC (Figure 2B) was ∼3 times higher over micro_In_2_O_3_/HZSM-5 compared to In_2_O_3_||HZSM-5. This enhancement in STY over micro_In_2_O_3_/HZSM-5 was further observed at 400 and 450 °C (see Figure S3). We hypothesize that the higher STY at the microscale could be related to the rate of CH_3_OH transfer from redox sites to the BAS. Accordingly, we estimated the rate of CH_3_OH advection (see Figure 2B, calculations described in Section S4.1 and Table S2) to be ∼10 times higher over micro_In_2_O_3_/HZSM-5 than In_2_O_3_||HZSM-5. Faster transfer and consumption of CH_3_OH over micro_In_2_O_3_/HZSM-5 likely shifts CH_3_OH synthesis equilibrium (from CO_2_ and H_2_, see Section S4.2) forward,^86^ consequently suppressing the formation of CO and increasing C_5+_ selectivity via methylation of lower olefins over BAS of HZSM-5.^38,70^

**Figure 3:**
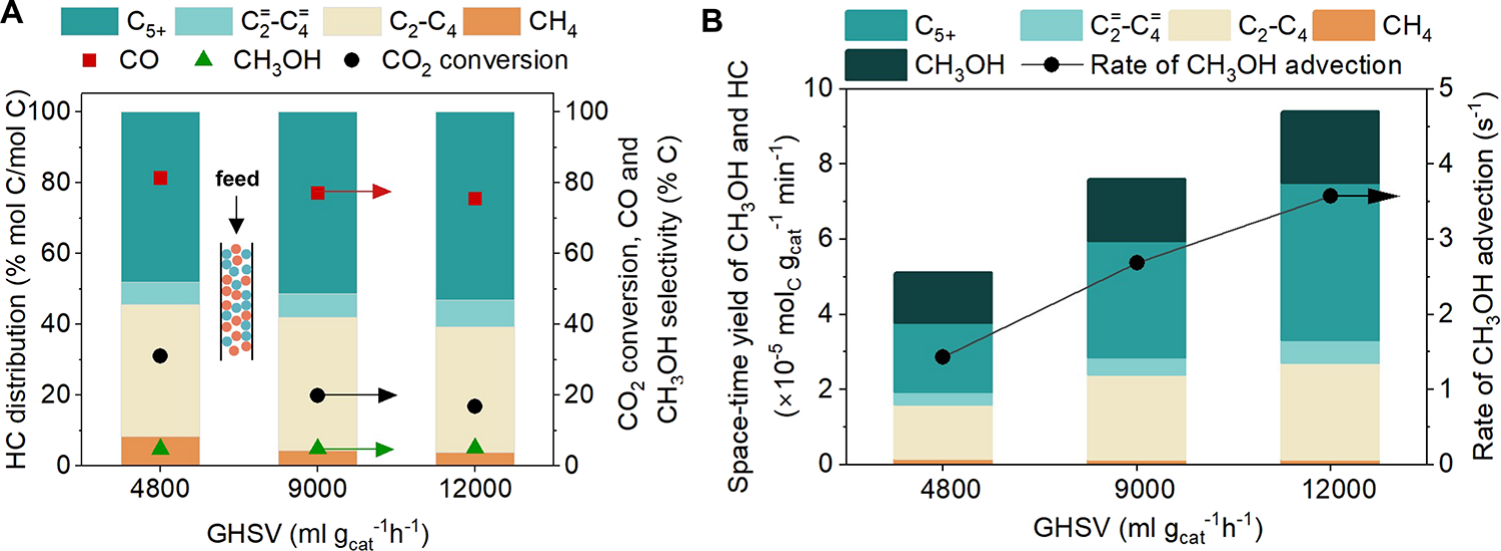
Effect of the rate of advective CH_3_OH transfer from redox sites of In_2_O_3_ and Bro̷nsted acid sites (BAS) of HZSM-5 on the catalytic activity. (A) Catalytic performance including hydrocarbon (HC) distribution (left axis), CO_2_ conversion, CO and CH_3_OH selectivity (right axis) during CO_2_ hydrogenation over micro_In_2_O_3_/HZSM-5 at different gas hourly space velocity (GHSV). (B) Space-time yield of CH_3_OH and HC over micro_In_2_O_3_/HZSM-5 (left axis) and rate of CH_3_OH advection (right axis) at different GHSV. Reaction conditions: 350 °C, 500 psig, GHSV 4800–12000 mL g_cat_^–1^ h^–1^, H_2_:CO_2_ ratio 3:1, In_2_O_3_:HZSM-5 mass ratio 1:1.

Since the rate of CH_3_OH advection depends on the gas flow rate (shown in Section S4.1), we further evaluated the catalytic performance of micro_In_2_O_3_/HZSM-5 at different GHSV (from 4800 to 12000 mL g_cat_^–1^ h^–1^) to explore how the rate of CH_3_OH transfer influences HC selectivity (see Figure 3A) and STY (see Figure 3B). Although CO_2_ conversion decreased by increasing GHSV, the STY of C_2+_ HC increased over micro_In_2_O_3_/HZSM-5 from 3.8 × 10^–5^ mol_C_ g_cat_^–1^ min^–1^ to 7.5 × 10^–5^ mol_C_ g_cat_^–1^ min^–1^ as the rate of CH_3_OH advection increased from 1.43 to 3.6 s^–1^. Concomitantly, the STY of unconverted CH_3_OH increased (1.3 × 10^–5^ mol_C_ g_cat_^–1^ min^–1^ to 1.9 × 10^–5^ mol_C_ g_cat_^–1^ min^–1^). We note that similar enhancements in C_2+_ HC STY were observed for In_2_O_3_||HZSM-5 (see Figure S5). Taken together, we deduce that the efficient transfer of CH_3_OH from redox to acid sites is crucial in achieving high HC STY and selectivity.

**Figure 4:**
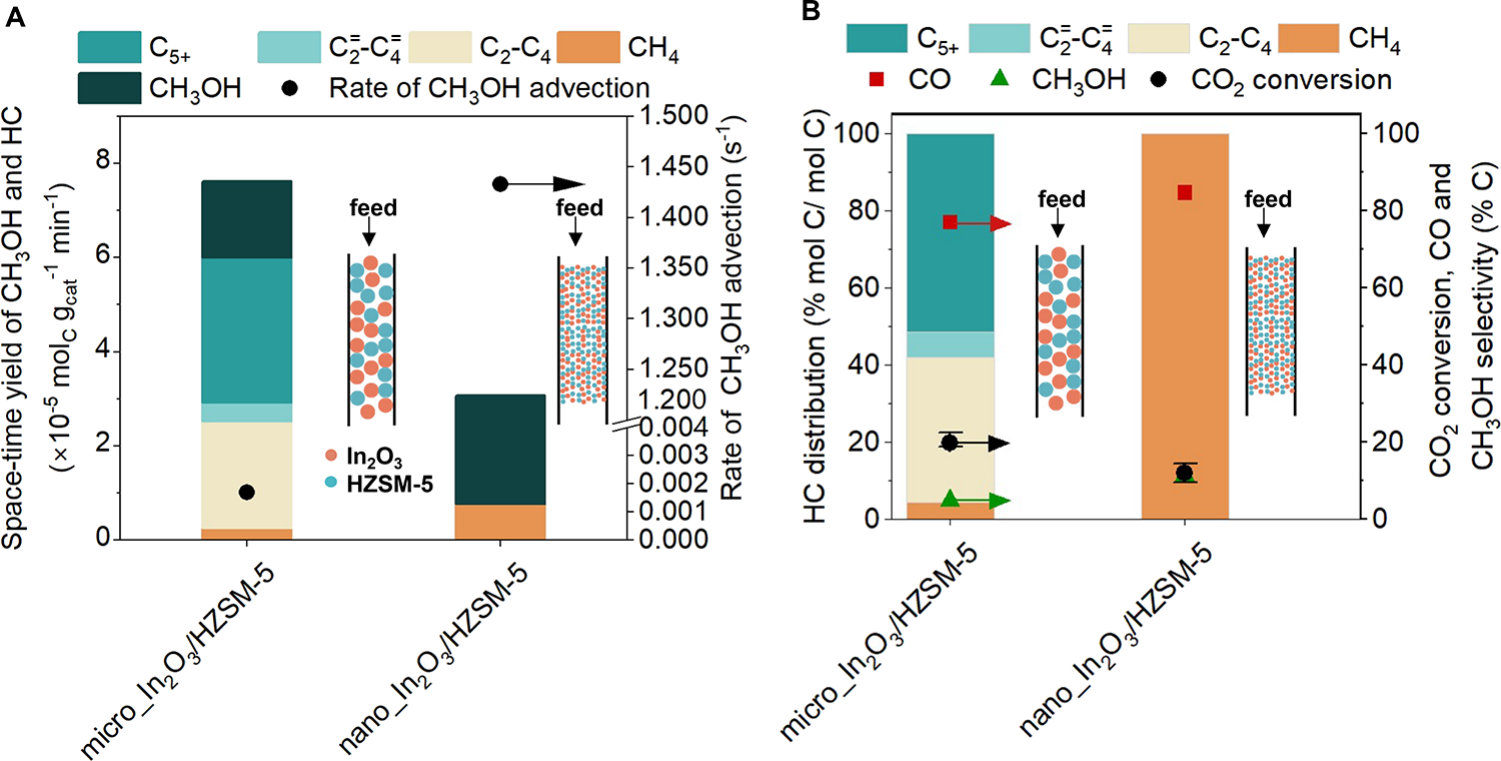
Influence of reducing the length scale between redox sites of In_2_O_3_and acid sites of HZSM-5 from microscale to nanoscale on the catalytic activity. (A) Space-time yield of CH_3_OH and hydrocarbon (HC) over micro_In_2_O_3_/HZSM-5 and nano_In_2_O_3_/HZSM-5 (left axis) and their respective rate of CH_3_OH advection (right axis); (B) catalytic performance including HC distribution (left axis), CO_2_ conversion, CO and CH_3_OH selectivity (right axis) during CO_2_ hydrogenation over micro_In_2_O_3_/HZSM-5 and nano_In_2_O_3_/HZSM-5. Reaction conditions: 350 °C, 500 psig, 9000 mL g_cat_^–1^ h^–1^, H_2_:CO_2_ ratio 3:1, In_2_O_3_:HZSM-5 mass ratio 1:1.

Interestingly, in the case of nanoscale admixture of In_2_O_3_ and HZSM-5, the combined STY of CH_3_OH and HC was lower (3.1 × 10^–5^ mol_C_ g_cat_^–1^ min^–1^) compared to micro_In_2_O_3_/HZSM-5, even though the rate of CH_3_OH advection was 3 orders of magnitude higher in nano_In_2_O_3_/HZSM-5 (see Figure 4A). Further, the sole hydrocarbon product was CH_4_ over nano_In_2_O_3_/HZSM-5. Additionally, the selectivity to unconverted CH_3_OH (11%) was found to be higher over nano_In_2_O_3_/HZSM-5, as compared to micro_In_2_O_3_/HZSM-5 (CH_3_OH selectivity 4.8%) (see Figure 4B), suggesting lower conversion of the CH_3_OH intermediate over HZSM-5 in nano_In_2_O_3_/HZSM-5.

### Structural, Textural, and Morphological Characterizations of Nano_In_2_O_3_/HZSM-5

To verify if the reduced reactivity of nano_In_2_O_3_/HZSM-5 was caused by any change in HZSM-5 structure, we conducted powder X-ray diffraction (PXRD) of nano_In_2_O_3_/HZSM-5, which exhibited characteristic peaks of HZSM-5 (see SI Figure S12A), suggesting that the crystallinity of HZSM-5 was largely retained in nano_In_2_O_3_/HZSM-5. Further, a comparison of scanning electron micrographs (Figure S10A) and transmission electron micrographs (TEM) of HZSM-5 (Figure S10B) with SEM and TEM of nano_In_2_O_3_/HZSM-5 (Figure S12B,C) revealed similar average particle size for HZSM-5 (∼500 nm) in the admixture, indicating no structural change to HZSM-5 in nano_In_2_O_3_/HZSM-5.

To further assess the structural and textural changes of HZSM-5 in nano_In_2_O_3_/HZSM-5, N_2_ physisorption was conducted for microscale and nanoscale admixtures. Both samples exhibited mesoporous type IV isotherm, which could be attributed to the mesoporous structure of In_2_O_3_ (Figure S13B).^91^ Although nano_In_2_O_3_/HZSM-5 exhibited a lower BET surface area and pore volume (202 m^2^/g, 0.25 cc/g) compared to micro_In_2_O_3_/HZSM-5 (256 m^2^/g, 0.29 cc/g), the micropore volume estimated from the t-plot was found to be similar (0.07 cc/g for micro_In_2_O_3_/HZSM-5 and 0.06 cc/g for nano_In_2_O_3_/HZSM-5) (see Table S3), suggesting the microporous structure of HZSM-5 was largely retained. However, the microporous area of nano_In_2_O_3_/HZSM-5 (130 m^2^/g) was found to be lower than micro_In_2_O_3_/HZSM-5 (172 m^2^/g), likely due to the coverage of the pore surface on HZSM-5 with In_2_O_3_.

Taking the PXRD patterns, SEM micrographs, and N_2_ physisorption data together, we posit that the structural integrity of HZSM-5 was largely retained in nano_In_2_O_3_/HZSM-5. We further note that the particle size of In_2_O_3_ in microscale admixtures is the same as sole In_2_O_3_ (∼20 nm Figure S10G), while in nano admixtures, the particle size is ∼10 nm (Figure S12C) likely induced by the mixing process. However, Lu et al. previously corroborated that the selectivity of CH_4_ remained similar (1.5–2.1% in the HC distribution) by varying In_2_O_3_ particle size from 7 to 28 nm. Hence, the increased CH_4_ selectivity over nano_In_2_O_3_/HZSM-5 was likely caused by the ion exchange of zeolitic BAS with In^δ+^ rather than any change in particle size of In_2_O_3_,^91^ or structural change of HZSM-5.

### Influence of Ion Exchange between Bro̷nsted Acid Sites (BAS) and In^δ+^ on Catalytic Activity

**Figure 5:**
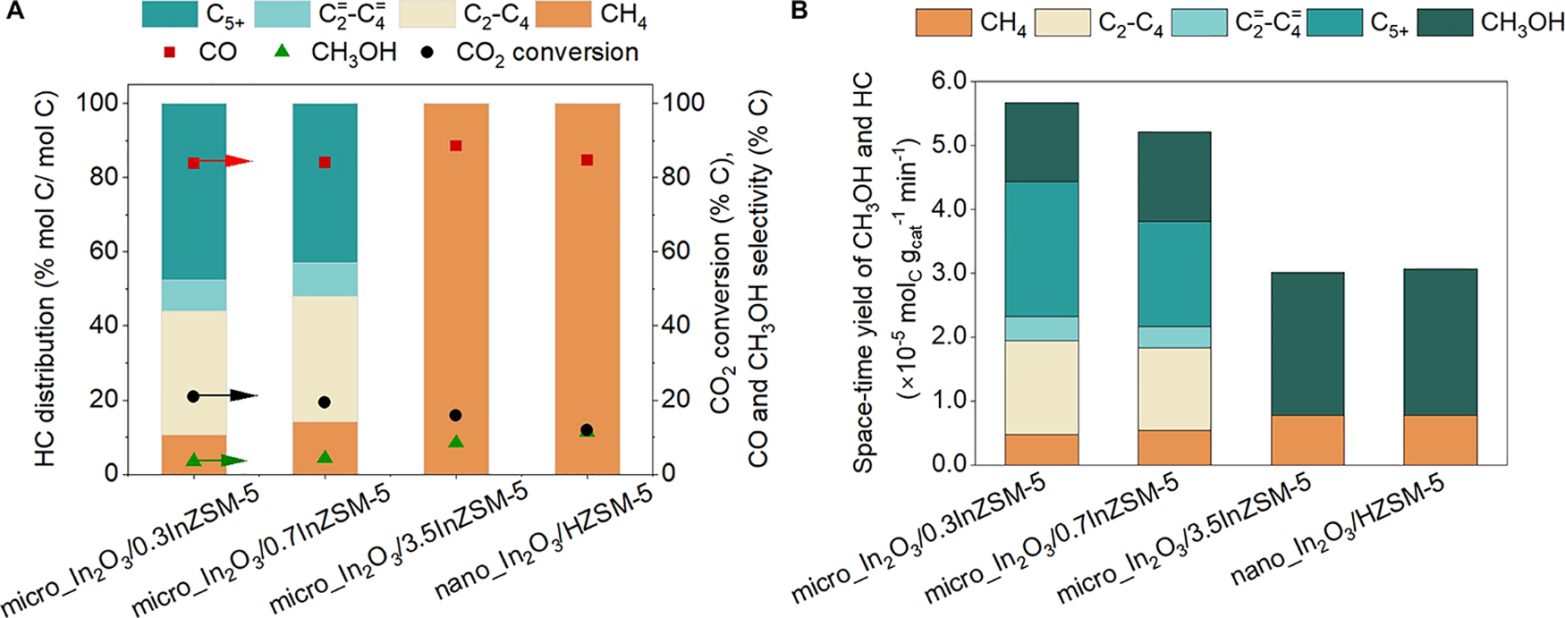
Effect of ion exchange of zeolitic H^+^with In^δ+^on catalytic activity. (A) Catalytic performance including hydrocarbon (HC) distribution (left axis), CO_2_ conversion, and CO and CH_3_OH selectivity (right axis) of micro_In_2_O_3_/0.3InZSM-5, micro_In_2_O_3_/0.7InZSM-5, micro_In_2_O_3_/3.5InZSM-5, and nano_In_2_O_3_/HZSM-5 during CO_2_ hydrogenation. (B) Space-time yield of CH_3_OH and HC over micro_In_2_O_3_/0.3InZSM-5, micro_In_2_O_3_/0.7InZSM-5, micro_In_2_O_3_/3.5InZSM-5, and nano_In_2_O_3_/HZSM-5. Reaction conditions: 350 °C, 500 psig, 9000 mL g_cat_^–1^ h^–1^, H_2_:CO_2_ ratio 3:1, In_2_O_3_:ZSM-5 mass ratio 1:1.

To probe whether the inhibited catalytic activity of nano_In_2_O_3_/HZSM-5 toward MTH was a result of ion exchange, zeolitic BAS were exchanged with In^δ+^ (δ likely to be 3) ions via incipient wetness impregnation (IWI) at three different In:Al molar ratios of 0.3, 0.7, and 3.5 (represented as *x*InZSM-5, where *x* = 0.3, 0.7, 3.5). The catalytic performance of microscale admixtures of In_2_O_3_ with *x*InZSM-5 (denoted as micro_In_2_O_3_/*x*InZSM-5) was studied as shown in Figure 5A. An increase in the In:Al ratio increased CH_4_ selectivity (10, 14, and 100% in HC distribution for *x* = 0.3, 0.7, and 3.5, respectively) indicating ion-exchange of BAS with In^δ+^ enhanced CH_4_ selectivity. Furthermore, the increase in In:Al ratio resulted in a marginally higher selectivity of unconverted CH_3_OH from 3.5 to 8.5%. Notably, the catalytic performance of micro_In_2_O_3_/3.5InZSM-5 was found to be similar to nano_In_2_O_3_/HZSM-5 (Figure 5A). Importantly, in these microscale admixtures (micro_In_2_O_3_/*x*InZSM-5 with *x* = 0.3, 0.7, and 3.5), the structural damage to HZSM-5 particles was unlikely as no powder mixing was employed. Further insights could be obtained on the effect of ion exchange by evaluating the STY of C_2+_ HC over micro_In_2_O_3_/*x*InZSM-5 (*x* = 0.3,0.7,3.5), as shown in Figure 5B. The STY of C_2+_ HC decreased (from 3.9 × 10^–5^ to 0 mol_C_ g_cat_^–1^ min^–1^) with the increasing In:Al ratio. Notably, for micro_In_2_O_3_/3.5In-ZSM-5 the ion exchange appeared complete as no C–C coupling products were observed, similar to nano_In_2_O_3_/HZSM-5 (see Figure 5A,B).

**Figure 6:**
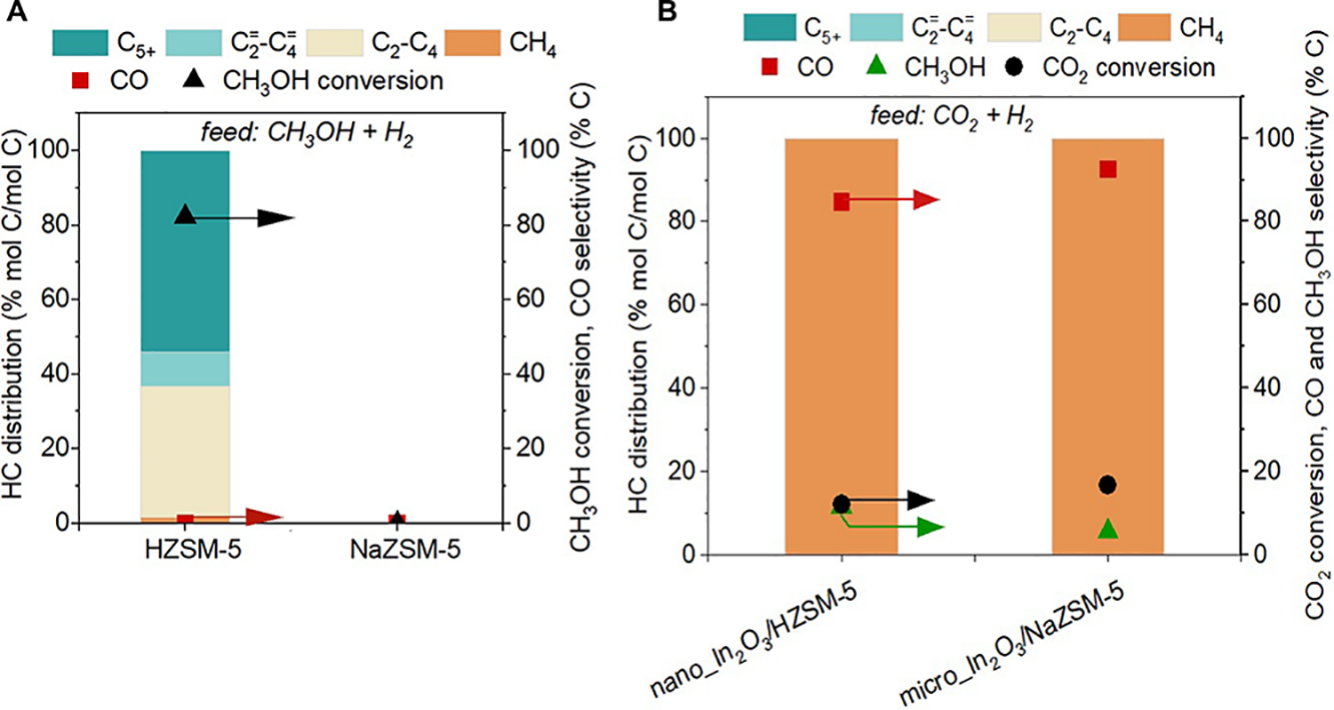
Inhibition of methanol to hydrocarbon (MTH) due to ion exchange of BAS with Na^+^. (A) Catalytic performance of NaZSM-5 including hydrocarbon (HC) distribution (left axis) and CH_3_OH conversion and CO selectivity (left axis) during MTH conversion confirmed complete ion exchange of BAS with Na^+^. Reaction conditions: CH_3_OH injection rate 0.012 mL min^–1^, 350 °C, GHSV of 9000 mL g_cat_^–1^ h^–1^ with H_2_:N_2_ ratio 3:1, total pressure 200 psi, partial pressure of CH_3_OH ∼6 psi; (B) Comparison between the catalytic performance of nano_In_2_O_3_/HZSM-5 and micro_ In_2_O_3_/NaZSM-5 during CO_2_ hydrogenation including HC distribution (left axis), CO_2_ conversion, CO and CH_3_OH selectivity (right axis). Reaction conditions: 350 °C, 500 psig, 9000 mL g_cat_^–1^ h^–1^, H_2_:CO_2_ ratio 3:1, In_2_O_3_:zeolite mass ratio 1:1.

To compare how the reaction would proceed without any BAS, we ion exchanged zeolitic BAS with Na^+^ (denoted as NaZSM-5 with Na:Al ratio of 1:1) and evaluated the catalytic performance of its microscale admixture with In_2_O_3_. Complete ion exchange of BAS with Na^+^ was confirmed by no MTH reactivity on NaZSM-5 as shown in Figure 6A. Notably, as anticipated, micro_In_2_O_3_/NaZSM-5 also showed no C–C coupling products, akin to nano_In_2_O_3_/HZSM-5 during tandem CO_2_ hydrogenation (Figure 6B).^92−94^ Taken together, our data suggests that in nanoscale admixture, the ion exchange of BAS with In^δ+^ causes a complete shutdown of MTH.

### Influence of Ion-Exchange on MTH and CH_3_OH Hydrodeoxygenation (HDO)

**Figure 7:**
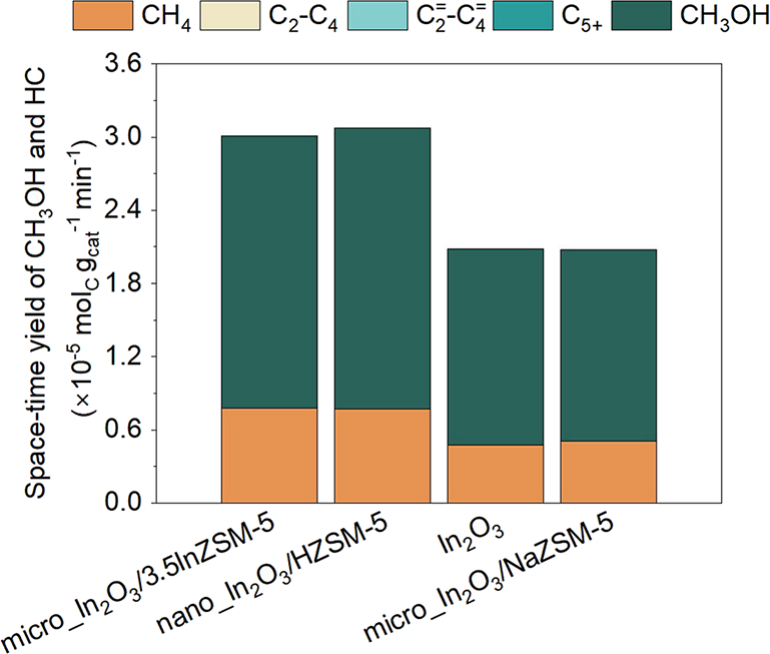
Space–time yield (STY) of hydrocarbon (HC) and CH_3_OH during CO_2_hydrogenation over micro_In_2_O_3_/3.5InZSM-5, nano_In_2_O_3_/HZSM-5, In_2_O_3,_and micro_In_2_O_3_/NaZSM-5. Higher STY for CH_3_OH and CH_4_ was observed over micro_In_2_O_3_/3.5InZSM-5 and nano_In_2_O_3_/HZSM-5, as compared to In_2_O_3_ and micro_In_2_O_3_/NaZSM-5. Reaction conditions: 350 °C, 500 psig, 9000 mL g_cat_^–1^ h^–1^, H_2_:CO_2_ ratio 3:1, In_2_O_3_:ZSM-5 mass ratio 1:1.

To investigate whether the ion-exchanged In^δ+^ species in micro_In_2_O_3_/3.5InZSM-5 and nano_In_2_O_3_/HZSM-5 have any influence on the reaction pathways, we compared their STY of C_1_ products with In_2_O_3_ and micro_In_2_O_3_/NaZSM-5. The STY toward C_1_ products were similar over micro_In_2_O_3_/3.5InZSM-5 and nano_In_2_O_3_/HZSM-5, however, higher than In_2_O_3_ and micro_In_2_O_3_/NaZSM-5, as shown in Figure 7. This further leads to the question of why the ion-exchanged In^δ+^ species enhanced STY of CH_3_OH and CH_4_ over nano_In_2_O_3_/HZSM-5 and micro_In_2_O_3_/3.5InZSM-5.

**Figure 8:**
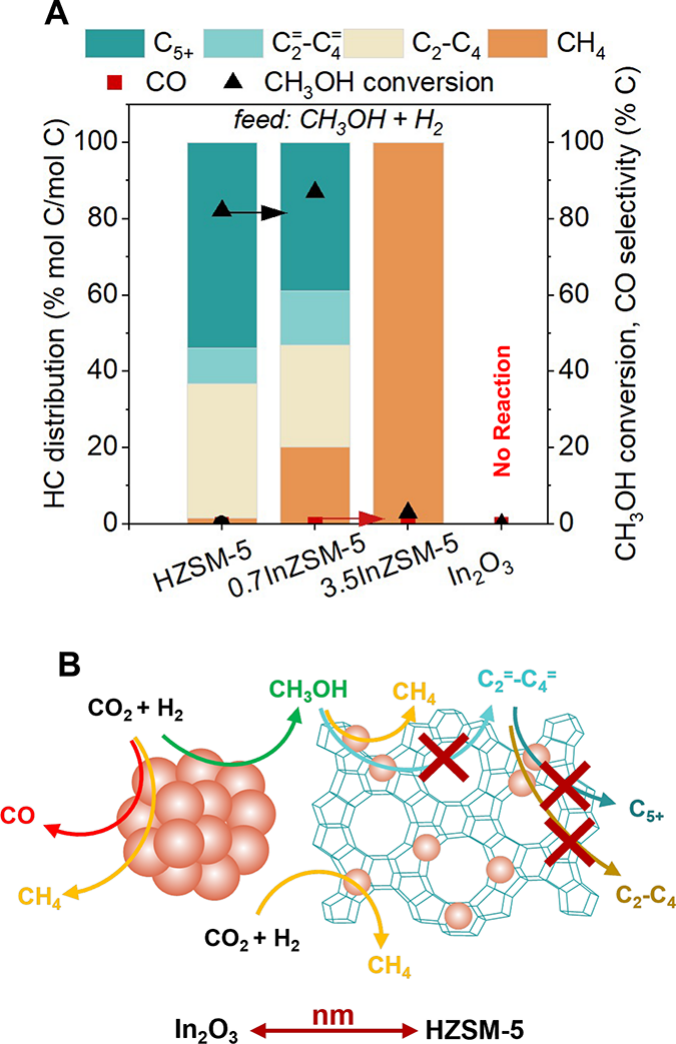
Ion exchange of zeolitic BAS with In^δ+^promotes methane formation. (A) MTH conversion over HZSM-5, 0.7InZSM-5, 3.5InZSM-5, and In_2_O_3_. Reaction conditions: CH_3_OH injection rate 0.012 mL min^–1^, 350 °C, GHSV of 9000 mL g_cat_^–1^ h^–1^ with H_2_:N_2_ ratio 3:1, total pressure 200 psi, partial pressure of CH_3_OH ∼6 psi. (B) Ion exchange of zeolitic H^+^ with In^δ+^ in nano_In_2_O_3_/HZSM-5 influences the reaction pathways during tandem hydrogenation of CO_2_.

For further investigation, we performed MTH over HZSM-5, 0.7InZSM-5, 3.5InZSM-5, and In_2_O_3_ (see Figure 8A). In_2_O_3_ alone did not exhibit any catalytic activity for MTH, while HZSM-5 showed 82% conversion of CH_3_OH with 1.6% CH_4_ selectivity in the HC distribution. Interestingly, 0.7InZSM-5 converted 87% CH_3_OH with a CH_4_ selectivity of 20%, and 3.5InZSM-5 converted 3% CH_3_OH at 100% CH_4_ selectivity, indicating the role played by ion-exchanged In^δ+^ species in CH_4_ formation during MTH. Several studies have reported that CH_3_OH hydrodeoxygenation (HDO) (CH_3_OH + H_2_ → CH_4_ + H_2_O) may occur in the presence of H_2_ during MTH.^95−97^ Therefore, we infer that CH_4_ is likely formed over ion-exchanged In^δ+^ species via CH_3_OH HDO,^95,98^ as In_2_O_3_ alone did not show any activity (see Figure 8A). It is to be noted that CH_3_OH HDO during MTH (in the absence of CO_2_) over In^δ+^ species is distinct from CO_2_ methanation observed over micro_In_2_O_3_/HZSM-5 (Figure 2A). Taken together, our data suggests that the increased CH_4_ STY during CO_2_ hydrogenation over nano_In_2_O_3_/HZSM-5 and micro_In_2_O_3_/3.5InZSM-5 (in Figure 7), is likely due to CH_3_OH HDO over the ion-exchanged In^δ+^ species (as depicted in the scheme of Figure 8B). However, the question remains on how CH_3_OH HDO occurs over ion-exchanged In^δ+^ sites.

### CH_3_OH Hydrodeoxygenation (HDO) over Ion-Exchanged In^δ+^ (In_2_O Moieties)

We performed spin-polarized density functional theory (DFT) calculations to assess the likely speciation of In species on BAS, the mechanism for CH_3_OH HDO, and the formation of CH_4_ over ion-exchanged In^δ+^ in HZSM-5 framework (see SI Section S4.3). To model the ion-exchanged In complex in ZSM5, an In_2_O moiety which is commonly documented in the literature was considered.^99^ We incorporated the In_2_O complex in the ZSM5 model by replacing the BAS. This represented the ion-exchanged extra framework moiety over the Al sites of ZSM-5. This catalyst model is hereafter referred to as In_2_O/ZSM-5 and is shown in Figure S9. The computed In–O–In stretching band at 361 cm^–1^ correlates with the In–O–In stretch at 382 cm^–1^ identified during Raman analysis (vide infra, Figure 10).

**Figure 9:**
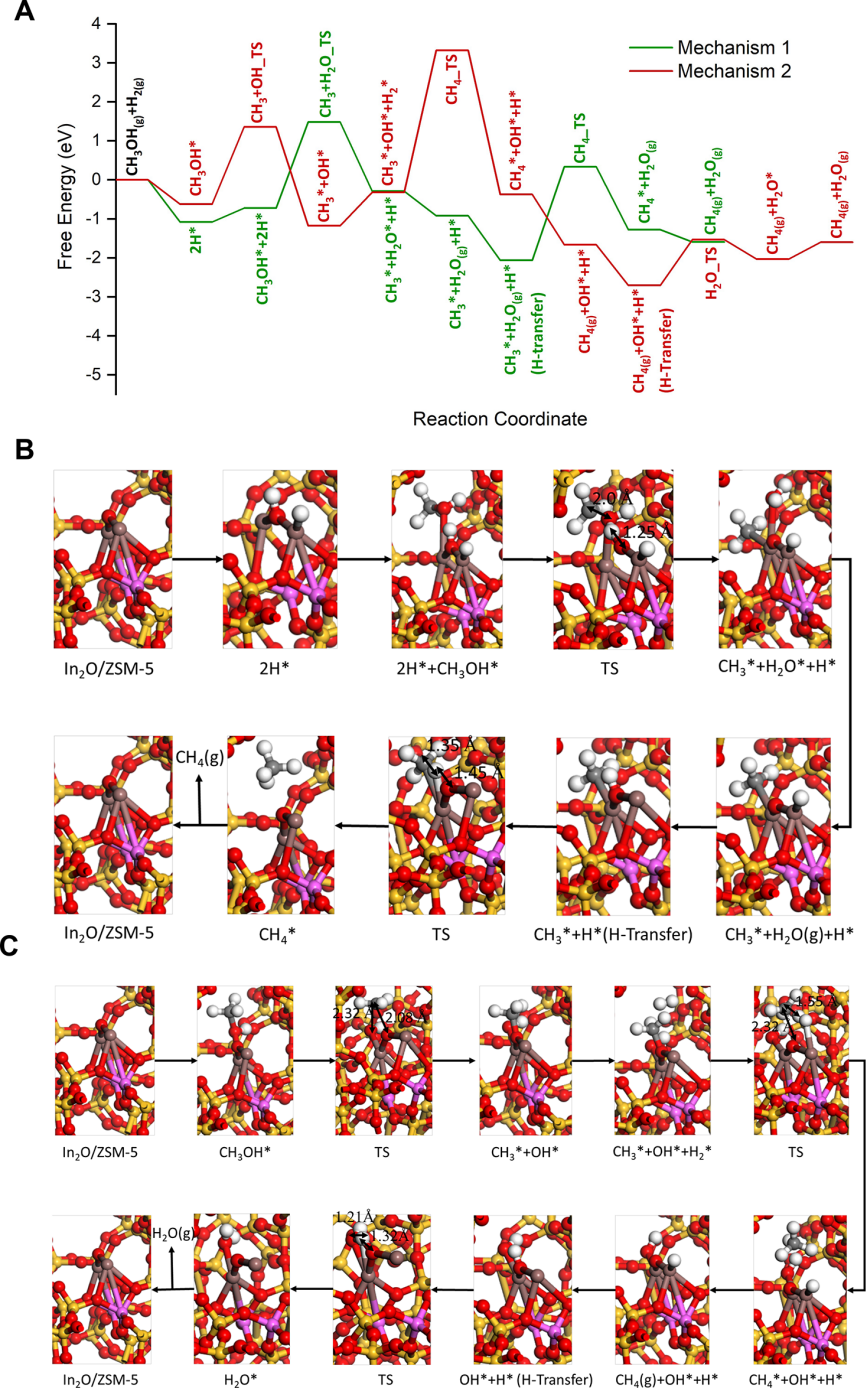
Free energy profiles for CH_3_OH hydrodeoxygenation (HDO) over In_2_O moieties inside HZSM5. Two mechanisms for HDO were investigated, indicated by green and red free energy profiles. (A) Mechanism 1 (green profile) assumed CH_3_OH adsorption on the In_2_O complex with preadsorbed and dissociated hydrogen forming an H–In–OH–In moiety. Mechanism 2 (red profile) assumed CH_3_OH adsorption on the pristine In_2_O complex. (B) Structures of the intermediates and transition states in mechanism 1. (C) Structures of the intermediates and transition states in mechanism 2. Red—O; magenta—Al; orange–yellow—Si; greyish mauve—In, gray—C, white—H.

Two mechanisms were investigated. The first mechanism assumed CH_3_OH adsorption on the In_2_O complex with preadsorbed and dissociated hydrogen forming an H–In–OH–In moiety. This assumption is reasonable considering the high H_2_ partial pressure in the feed. The free energy profile along this mechanism is shown in green color in Figure 9A and the structures of the intermediates and transition states are shown in Figure 9B. The second mechanism assumed CH_3_OH adsorption on the pristine In_2_O complex. The free energy profile along this mechanism is shown in red color in Figure 9A and the structures of the intermediates and transition states are shown in Figure 9C.

The heterolytic dissociation of H_2_ on the In_2_O moiety is highly exergonic (Δ*G*_ads_ = −1.08 eV). In mechanism 1, CH_3_OH underwent a bridged hydroxyl O_br_-H assisted deoxygenation step to form water and CH_3_* with an activation-free energy barrier of 2.21 eV (Δ*G*_rxn_ = 0.44 eV). Following the desorption of the formed water, the In–H migrated to the bridged oxygen as this was highly favorable. Hydrogenation of the methyl species and formation of CH_4_ had a high activation free energy barrier of 2.39 eV (Δ*G*_rxn_ = 0.78 eV). In mechanism 2, CH_3_OH underwent a direct C–O bond cleavage at the In site in the In_2_O moiety to form a methoxy species (CH_3_–O_br_) and In–OH. This step had a slightly lower activation-free energy barrier of 1.98 eV compared to the proton-assisted step in mechanism 1. A concerted H_2_ dissociation and CH_3_ hydrogenation step to form methane followed, with a very high activation-free energy barrier of 3.64 eV. Upon desorption of the formed CH_4_, the In–H migrated to the bridged oxygen. The subsequent formation of water had an activation-free energy barrier of 1.17 eV. Analysis of the free energy profiles along both these mechanisms suggests that mechanism 1 with the Obr-H-assisted hydrodeoxygenation of CH_3_OH is kinetically more favorable. However, the kinetically relevant step in this mechanism appears to be the hydrogenation of the methyl species. The high activation-free energy barriers computed here indicate that the CH_3_OH HDO at the In exchanged sites is likely to be slow. This is consistent with the low conversion of CH_3_OH on the ZSM-5 in the nano_In_2_O_3_/HZSM-5 and the overall lower activity of this catalyst post ion exchange (Figures 5 and 6).

**Figure 10:**
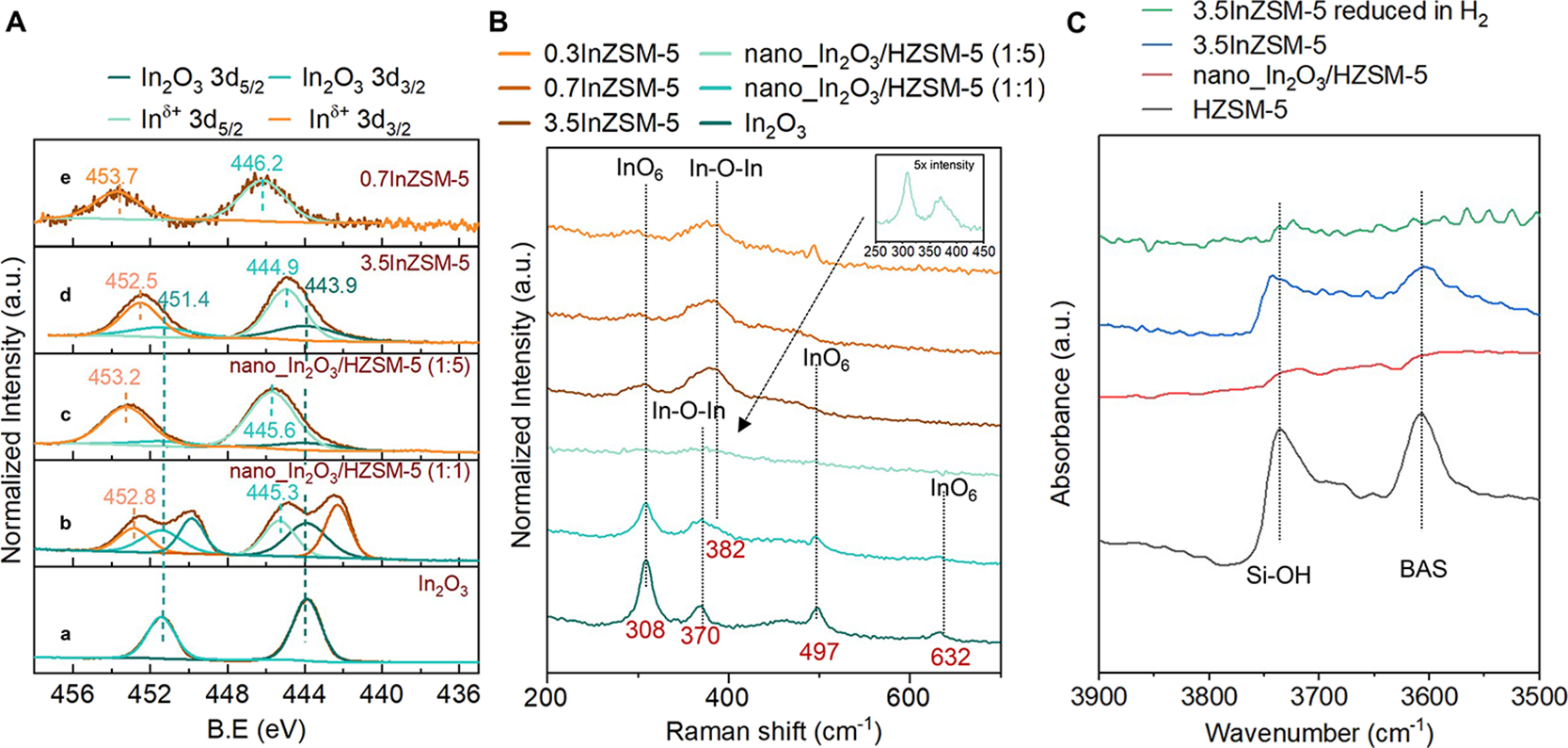
Probing the ion exchange of zeolitic H^+^with In^δ+^via spectroscopic techniques. (A) XPS of In (3d) energy region for In_2_O_3_, nano_In_2_O_3_/HZSM-5 (1:1), nano_In_2_O_3_/HZSM-5 (1:5), 3.5InZSM-5, and 0.7InZSM-5; (B) Raman spectra for In_2_O_3_, nano_In_2_O_3_/HZSM-5 (1:1), nano_In_2_O_3_/HZSM-5 (1:5), 0.3InZSM-5, 0.7InZSM-5, and 3.5InZSM-5. The inset on the top right shows zoomed-in spectra for nano_In_2_O_3_/HZSM-5 (1:5) at 5× intensity. (C) Fourier transform infrared (FTIR) spectra of pristine HZSM-5 (black), 3.5InZSM-5 (blue), and nano_In_2_O_3_/HZSM-5 (1:1) (red).

Although our DFT calculations suggested that In_2_O moieties likely form due to ion exchange at the nanoscale, we further investigated the oxidation state of In^δ+^ species via X-ray photoelectron spectroscopy (XPS). Figure 10A shows XPS spectra of pristine In_2_O_3_, nano_In_2_O_3_/HZSM-5 (1:1), 0.7In-ZSM-5, 3.5InZSM-5, and, in addition, nano_In_2_O_3_/HZSM-5 (1:5), which has an equivalent In:Al ratio as 3.5InZSM-5 (see PXRD in Figure S21). No peaks corresponding to metallic In were observed for all samples, consistent with the PXRD patterns in Figures S12A and S14A. Bulk In_2_O_3_ (spectra **a**) exhibited characteristic peaks at binding energy (B.E.) values of 443.9 and 451.4 eV for In 3d_5/2_ and In 3d_3/2_, respectively.^99−101^ Interestingly for nano_In_2_O_3_/HZSM-5 (1:1) (spectra **b**), in addition to In_2_O_3_ peaks at 443.9 and 451.4 eV, higher B.E. peaks at 445.3 and 452.8 eV,^99,102^ and lower B.E. peaks at 442.3 and 449.8 eV were observed. For nano_In_2_O_3_/HZSM-5 (1:5) (spectra **c**), peaks corresponding to In_2_O_3_ (443.9 and 451.4 eV) and at higher binding energies (445.6 and 453.2 eV) were observed with no peaks at lower B.E. Therefore, we hypothesize that the lower B.E. peaks seen in the XPS of nano_In_2_O_3_/HZSM-5 (1:1) (spectra **b**) could be associated with oxygen vacancies/partially reduced In_*x*_O_*y*_. Considering that the nano_In_2_O_3_/HZSM-5 (1:5) has a much lower loading of In_2_O_3_ than nano_In_2_O_3_/HZSM-5 (1:1), we infer that the absence of a lower B.E. peak could be due to low concentration of oxygen vacancies that are beyond the detection limit of the XPS.

Among ion-exchanged *x*InZSM-5 samples, 3.5InZSM-5 (spectra **d**) showed peaks corresponding to bulk In_2_O_3_ (443.9 and 451.4 eV) and at higher B.E. (444.9 and 452.5 eV). For 0.7InZSM-5 (spectra **e**), no detectable peaks corresponding to In_2_O_3_ were observed. However, the peaks shifted further to higher B.E. (446.2 and 453.7 eV), as compared to 3.5InZSM-5. Due to the low signal-to-noise ratio (i.e., low resolution), XPS was not shown for 0.3InZSM-5 samples.

Overall, the XPS of ion-exchanged *x*InZSM-5 (*x* = 0.7,3.5) and nanoscale admixtures (nano_In_2_O_3_/HZSM-5 (1:1) and nano_In_2_O_3_/HZSM-5 (1:5)) exhibited peaks at higher B.E., as compared to In_2_O_3_. This observation is consistent with previous studies where a shift toward higher B.E. (445.5 and 453 eV) was attributed to the ion exchange of BAS with In^δ+^.^76,77,99^ Therefore, these higher B.E. peaks could be associated with ion-exchanged In^δ+^ species interacting with the HZSM-5 framework likely leading to In_2_O moieties.^99,103−105^

Further evaluation of ion exchange was conducted by Raman spectroscopy on pristine In_2_O_3_, nano_In_2_O_3_/HZSM-5 (1:1), nano_In_2_O_3_/HZSM-5 (1:5), 0.3InZSM-5, 0.7InZSM-5, and 3.5InZSM-5, shown in Figure 10B. In agreement with XPS and PXRD, no samples exhibited bands for metallic In at 250 and 620 cm^–1^ (RRUFF ID: R060771.2),^106,107^ and cubic In(OH)_3_ at 137, 204, 356, 390, and 659 cm^–1^.^108,109^ Pure In_2_O_3_ exhibited bands at 308, 497, and 632 cm^–1^, which is attributed to the InO_6_ vibration of the cubic phase of In_2_O_3_, consistent with PXRD patterns. The band at the Raman shift of 370 cm^–1^ can be assigned to the stretching vibration of the In–O–In bonds.^108,110^

Raman spectra of nano_In_2_O_3_/HZSM-5 (1:1) showed bands at 307, 497, and 632 cm^–1^ for InO_6_ octahedra,^108,110^ along with the In–O–In band at 370 cm^–1^. Interestingly, a shoulder appeared at ∼382 cm^–1^, which was not exhibited by bulk In_2_O_3_. For nano_In_2_O_3_/HZSM-5 (1:5), the Raman spectra had low resolution due to the lower loading of In_2_O_3_, however, a similar shoulder was visible (see inset in Figure 10B).

For ion-exchanged 0.3InZSM-5, 0.7InZSM-5, and 3.5InZSM-5, the InO_6_ bands at 308, 497, and 632 cm^–1^ were reduced while the band for In–O–In broadened and shifted toward the higher wavenumber of 382 cm^–1^. This shift to a higher wavenumber is consistent with previous literature where the band shifted to 400 cm^–1^ for In^δ+^ ion-exchanged HZSM-5.^108^ Taken together, the band at 370 cm^–1^ was likely associated with oxygen vacancies, while the broadening and shifting of this band to 382 cm^–1^ was likely related to the ion exchange of BAS with In^δ+^. As mentioned earlier, this is consistent with the In_2_O moieties (Figure 9) inside the zeolite as suggested by DFT calculations.

Taking the findings from Raman and XPS together, we postulate that the Raman band at 382 cm^–1^ (Figure 10B), seen in all samples containing HZSM-5 but not seen for In_2_O_3_, is related to the ion-exchanged In^δ+^ species (likely In_2_O moieties). This observation further aligns with the higher B.E. peaks seen in the XPS by all samples containing HZSM-5 (Figure 10A) indicating the interaction of In^δ+^ species with the HZSM-5 at a higher B.E.

The disappearance of peaks corresponding to the BAS of HZSM-5 at a wavenumber of 3605 cm^–1^ in the Fourier transform infrared (FTIR) spectroscopy is consistent with the solid-state ion-exchange (SSIE) of BAS with In^δ+^ species (as shown in Figure 10C). We suggest that the In^δ+^ species may not be In^+^, as the In^+^ peaks would have shifted to a lower B.E. than bulk In_2_O_3_ (443.9 and 451.4 eV for In 3d_5/2_ and In 3d_3/2_, respectively). Interestingly, in ion-exchanged 3.5InZSM-5, the ion exchange (via IWI) appeared incomplete, as revealed by FTIR spectroscopy (see the blue spectrum in Figure 10C) after in situ calcination at 400 °C under air. However, upon reduction (at 400 °C with an H_2_:N_2_ ratio of 1:1) the ion exchange was complete as seen from the complete disappearance of peaks corresponding to silanol (Si–OH) and BAS of HZSM-5 at wavenumbers of 3740 and 3605 cm^–1^, respectively (green spectrum). Therefore, under the reductive reaction conditions, the BAS of zeolites was likely completely ion-exchanged with cationic In^δ+^ species. Under reductive conditions similar to our work, Xie et al. have suggested that reducible metal oxides can migrate from zeolite surfaces to micropore channels.^77^ Furthermore, under the harsh reaction conditions used in our study, metal cations formed from metal oxides could have higher thermal mobility, accelerating ion exchange under reductive conditions.^112−114^ It has been further suggested that under a reductive environment, InO^+^ could convert to In^+^.^99,115^ From our data, we conclude that these ion-exchanged species inhibit C–C coupling by reducing HZSM-5 acidity and promoting CH_4_ formation via CH_3_OH hydrodeoxygenation (HDO).

## Conclusions

Overall, the microscale (∼300 μm) placement between the redox sites of In_2_O_3_ and Bro̷nsted acid sites (BAS) of HZSM-5 exhibited enhanced catalytic activity (STY of CH_3_OH and HC ∼3× higher at 350 °C) as compared to milliscale (∼10 mm), which was attributed to the efficient transfer and conversion of CH_3_OH intermediate (rate of CH_3_OH advection was 10-fold faster at microscale than milliscale). Although the transfer of CH_3_OH was more efficient at nanoscale placement (∼300 nm), the occurrence of solid-state ion exchange (SSIE) between Bro̷nsted acid sites (H^+^) of HZSM-5 and In^δ+^ ions from In_2_O_3_ was observed, which completely inhibited methanol to hydrocarbons (MTH) reaction and promoted only CH_4_ formation via CH_3_OH hydrodeoxygenation (HDO) likely on In_2_O moieties inside HZSM-5. Based on our data, we infer that the rate of CH_3_OH advection and the prevention of ion exchange between zeolitic BAS and In^δ+^ from In_2_O_3_ are the key factors in achieving catalytic synergy in the bifunctional In_2_O_3_/HZSM-5 system. Our work enriches the understanding of the structure–activity relationship of bifunctional oxide-zeolite systems and offers valuable insights into the synergistic design of tandem catalysts.

## Supporting Information Available

Computed distances between redox and BAS, CH_3_OH advection rates, additional reactivity, N_2_ physisorption isotherms and BET surface areas, TEM, SEM, Raman, and XPS, and detailed mass balances of the tandem reactions referenced in the text (PDF)
