# Supplementary material for: Intermediate Transfer Rates and Solid-State Ion Exchange are Key Factors Determining the Bifunctionality of In2O3/HZSM-5 Tandem CO2 Hydrogenation Catalyst
Source: ACS Sustain Chem Eng. 2024 Mar 18;12(13):5197–210. doi: 10.1021/acssuschemeng.3c08250 (PMC10988559; doi:10.1021/acssuschemeng.3c08250)
Supplement: Supplementary file 1 — sc3c08250_si_001.pdf [file sc3c08250_si_001.pdf]

Supporting Information

Intermediate Transfer Rates and Solid-State Ion Exchange are Key Factors Determining the Bifunctionality of In_2O_3/HZSM-5 Tandem CO_2 Hydrogenation Catalyst

*Fatima Mahnaz[1], Jasan Robey Mangalindan[1], Balaji C. Dharmalingam [2], Jenna Vito[1], Yu-Ting Lin[1], Mustafa Akbulut[1], Jithin John Varghese[2] & Manish Shetty[1]**

[1]Artie McFerrin Department of Chemical Engineering, 100 Spence Street, Texas A&M University College Station, TX 77843, USA.

[2]Department of Chemical Engineering, Indian Institute of Technology Madras, Chennai, Tamil Nadu 600036 India.

*Email. manish.shetty@tamu.edu

Number of pages – 55
Number of tables – 14
Number of figures – 22

S1. Catalyst characterization methods

Powder X-ray Diffraction (PXRD). The PXRD patterns were acquired using a Rigaku Miniflex II X-ray instrument equipped with Cu-Kα radiation (λ=1.5406 Å). The scanning range was set from 5 to 75°, with a step rate of 0.02 and a scan rate of 1°/min. The PXRD patterns of In_2O_3, micro_In_2O_3/HZSM-5, and nano_In_2O_3/HZSM-5 were normalized with respect to the In_2O_3 peak at 30.8°, while PXRD patterns for HZSM-5 were normalized with respect to the peak at 7.9°.

N_2 physisorption. Surface area measurements were performed using the Anton Paar Autosorb iQ-C-MP EPDM automated gas sorption analyzer. The surface area analysis was carried out using nitrogen physisorption at 77 K, and the resulting adsorption-desorption isotherms were analyzed using the Brunauer-Emmett-Teller (BET) theory.[1] For physisorption, a quantity of 10-150 mg of the catalyst was placed in a 6 mm glass cell bulb (without a rod). The sample underwent an initial outgassing process at 350°C for a duration of 480 minutes. Nitrogen physisorption isotherms were then conducted, encompassing 75 adsorption and 20 desorption points ranging from p/p_o values of 0.025 to 0.995. The isotherm data were subjected to BET analysis, specifically utilizing the adsorption data points ranging from p/p_o values of 0.005 to 0.3. Total pore volume was calculated based on the assumption that at relative pressures near unity, the pores completely filled with liquid following the equation below:[2]

$$V_{liq}=\frac{P_a V_{ads} V_m}{RT} \tag{S1}$$

The micropore area and volume were calculated using the t-plot method using the deBoer thickness equation (see below) using adsorption data points ranging from p/p_o values of 0.2 to 0.5. All zeolite containing samples contained a positive y-intercept on the t-plot indicating the presence of micropores, whereas the bulk In_2O_3 sample's t-plot passed through the origin confirming it to be mesoporous in nature.

$$t=\left[\frac{13.99}{\log\left(P_0/P\right)+0.034}\right]^{\frac{1}{2}} \tag{S2}$$

The t-plot is a graph of volume of gas adsorbed at STP (V_{ads}^{STP}) vs the layer thickness (t). The slope of this line (s) is related to the total surface area of the pores given by the equation below. It then follows that the micropore surface area (S_{MP}) is the difference between the BET surface area and S_t.

$$S_t\ (m^2/g) = \frac{V_{ads}^{STP} \times 15.47}{t} = s \times 15.47 \quad (S3)$$

$$S_{MP} = S_{BET} - S_t \quad (S4)$$

For samples without micropores, there is good agreement between S_{BET} and S_t (such is the case for bulk In_2O_3). The micropore volume is related to the intercept (i) of this plot given by the following equation.

$$V_{MP} = i \times 0.001547\ (cm^3) \quad (S5)$$

Details on BET calculation parameters (slope and intercept) and micropore volume calculations are given in **Table S3**.

X-ray photoelectron spectroscopy (XPS). XPS measurements were conducted at the Materials Characterization Facility (MCF) located at Texas A&M University, utilizing the EnviroESCA XPS instrument.

The XPS spectra were analyzed using CasaXPS software (version 2.3.16 PR 1.5). Shirley background was selected and the C 1s spectra for adventitious carbon were used for charge correction by assigning the C-C, C-H component binding energy to 284.8 eV (See **Table S5** for additional assignments and constraints). Peak deconvolution was done using a Gaussian-Lorentzian (GL30) line shape. The In^{3+} was assigned to 443.9 eV based on the bulk In_2O_3 spectra with a peak splitting placement of 7.54 eV and spin-orbit splitting ratio of 3:2 ($3d_{5/2}$:$3d_{3/2}$) for the 3d spectrum. InZSM-5 spectra were then fitted with additional components, utilizing the same splitting constraints, to account for peak broadening seen with zeolite samples compared to bulk In_2O_3 (**Table S6**).

Scanning electron microscopy (SEM). SEM for all samples was done without any coating. SEM imaging for 0.7InZSM-5 and 3.5InZSM-5 was performed using the Tescan LYRA-3 Model GMH Focused Ion Beam Microscope at 39400x and 70100x magnification, respectively, with an operational voltage of 7kV. SEM of 0.3InZSM-5 was done in JEOL JSM-7500F at 15000x magnification and 5kV, and SEM of nano_In_2O_3/HZSM-5 was done using FERA-3 Tescan Model GMH Focused Ion Beam Microscope at 54000x magnification and 7kV.

Transmission electron microscopy (TEM). TEM analysis was conducted using the FEI TECNAI G2 F20 CRYO FE-TEM instrument. Prior to TEM, approximately 5 mg of powder sample was added to 500 mg of Isopropanol (IPA, 70% v/v, Millipore Sigma, Burlington, Massachusetts, US) and sonicated for 2 min in a water bath at room temperature. A drop of the sonicated mixture was then added to a copper grid of 200 mesh (formvar coating thickness 10nm and carbon coating thickness 1 nm, purchased from Electron Microscopy Sciences) and dried overnight in a dryer at 50 °C. Particle size distribution was done manually as shown in **Figure S9**.

Raman Spectra. Raman spectra were collected using a Thermo Scientific DXR Raman microscope, equipped with a 780 nm diode laser, a 10x objective lens, a Rayleigh rejection filter, a diffraction grating with a resolution of 4.7-8.7 cm^{-1}, a 25 µm pinhole, and a black-illuminated charged-coupled device (CCD) detector.

Fourier Transform Infrared (FTIR) Spectra. FTIR spectra of **Figure 9C** were collected in Nicolet is50 equipped with a deuterated triglycine sulfate (DTGS) detector, with spectral resolution 8 cm^{-1}. Before collecting the spectra, the samples were pressed into a self-supported wafer (~8 mg) and treated *in-situ* using a Harrick HTC transmission cell at 450 °C for 5 h, followed by an ultra-high vacuum of 10^{-4} mbar for 5 h. IR spectra were then obtained at 150 °C. The spectra were analyzed in Thermo Scientific Omnic Series Software. All the spectra presented in this work were normalized with ~8 mg sample mass and the band intensity at 802 cm^{-1} (for Si-O-Si bond of HZSM-5).

S2. Estimation of the distance between the active sites of In_2O_3 and HZSM-5 in bifunctional admixtures

S2.1. In_2O_3||HZSM-5 and HZSM-5||In_2O_3

The average distance (D) between In_2O_3 and HZSM-5 particles in In_2O_3||HZSM-5 and HZSM-5||In_2O_3 was estimated to be the length between the midpoints of the In_2O_3 bed and HZSM-5 bed with bed heights H_{In2O3} and $H_{HZSM\text{-}5}$, respectively. In between the two beds was Al_2O_3 which had a height of H_{Al2O3} = 3 mm.

$$D = \frac{H_{In2O3}}{2} + H_{Al2O3} + \frac{H_{HZSM-5}}{2} \quad (S6)$$

The bed heights were calculated based on the mass (m), packed bed density of the catalyst (ρ), and inner cross-sectional area of the reactor tube (A). The density of the catalyst bed was approximately 1.6 g/cm^3.

$$D = \frac{m_{In2O3}}{2 \cdot \rho_{In2O3} \cdot A} + H_{Al2O3} + \frac{m_{HZSM-5}}{2 \cdot \rho_{HZSM-5} \cdot A} \quad (S7)$$

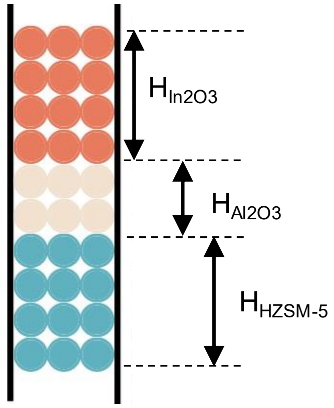

Figure S1: Estimation of distance between In_2O_3 and HZSM-5 particles in In_2O_3||HZSM-5 admixture.

S2.2. micro_In_2O_3/HZSM-5 and nano_In_2O_3/HZSM-5

We used an iterative method to estimate the distance between In_2O_3 and HZSM-5 particles in micro_In_2O_3/HZSM-5 and nano_In_2O_3/HZSM-5 admixtures. Specifically, the two catalyst particles can be considered ideal spheres of two radii with a homogeneous distribution of active sites. The radius of the In_2O_3 particle is noted as R1 and the radius of the HZSM-5 particle is R2.

For micro_In_2O_3/HZSM-5, we used the radius of the granule size of In_2O_3 and HZSM-5 as R1 and R2, whereas for nano_In_2O_3/HZSM-5, we used the particle size of In_2O_3 and HZSM-5 from **Figure S12B, S12C** as R1 and R2, respectively. Each granule/particle can be considered an ensemble of active units. To begin, we utilized Python to construct a model of the two granules/particles. We treated each granule/particle as a composite of multiple spherical shells, forming concentric layers. Let us denote the sets of shell radii as S_{R_1} and S_{R_2}, respectively. For each $r_1 \in S_{R_1}$, we first determine the number of active sites n_{r_1} on that shell using the distribution function,[3]

$$F\,(r) = \frac{r^2}{1+e^{-r}} \tag{S8}$$

Similarly, we pick a shell radius on R2, that is, $r_2 \in S_{R_2}$ and compute the number of active sites on that shell n_{r_2}. We assume that both shells are symmetric with respect to the z-axis. Next, for each active site on the shell with radius r_2, we first compute the Euclidean distance to all n_{r_1} active sites on R1. Let us denote this sum as $dist_1$, which is the total Euclidean distance from the first active site on R2 with radius r_2 to all the n_{r_1} active sites on R1 with radius r_1. We repeat this process for all n_{r_2} active sites on R2. After that, we compute the normalized average distance for a fixed (r_1, r_2) as follows:

$$d(r_1, r_2) = \frac{\left(\sum_{i=1}^{n_{r_2}} \frac{dist_i}{n_{r_1}}\right)}{n_{r_2}} \tag{S9}$$

Finally, we compute a weighted average distance, D as follows:

$$D = \frac{\sum_{r_1 \in S_{r_1}} \sum_{r_2 \in S_{r_2}} F(r_1) \cdot F(r_2) \cdot d(r_1, r_2)}{\sum_{r_1 \in S_{r_1}} \sum_{r_2 \in S_{r_2}} F(r_1) \cdot F(r_2)} \tag{S10}$$

Python code for the estimation of distance between active sites is shown at the end of SI.

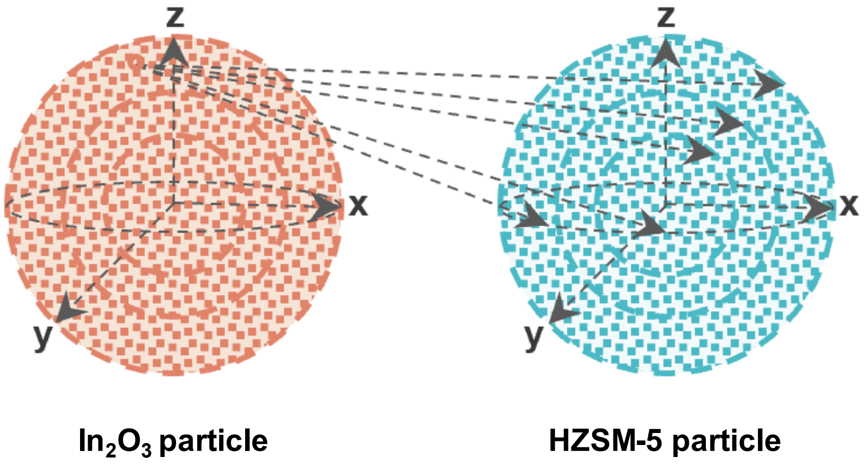

Figure S2: Estimation of distance between the active sites of In_2O_3 and HZSM-5 micro_In_2O_3/HZSM-5 and nano_In_2O_3/HZSM-5 admixtures.

Table S1: Results from distance estimation between the active sites in In_2O_3||HZSM-5, micro_In_2O_3/HZSM-5, and nano_In_2O_3/HZSM-5 admixtures.

**Type of arrangement**	**Size of HZSM-5 granule/particle**	**Size of In_2O_3 granule/particle**	**Range of distance between active sites**	**Weighted average distance between active sites**
**In_2O_3||HZSM-5**	400 μm	400 μm	3-16 mm	9.6 mm
**micro_In_2O_3/HZSM-5**	400 μm	400 μm	207-325 μm	303 μm
**nano_In_2O_3/HZSM-5**	500 nm	20 nm	253-334 nm	315 nm

S3. Reactivity studies

S3.1. Catalytic synergy exhibited at different reaction temperatures by micro_In_2O_3/HZSM-5

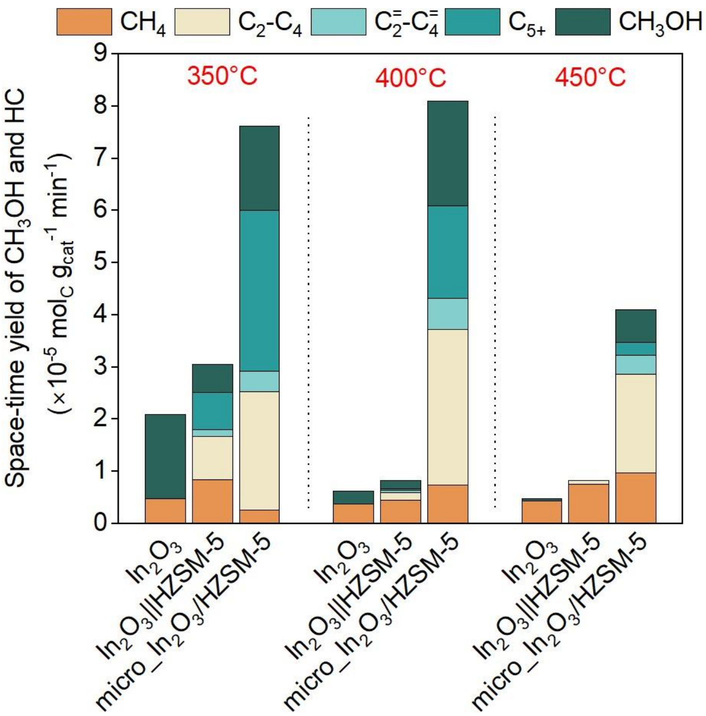

Figure S3: Catalytic synergy exhibited at different reaction temperatures by micro_In_2O_3/HZSM-5 in terms of the space-time yield of CH_3OH and HCs, compared to In_2O_3 and In_2O_3||HZSM-5. Reaction conditions: 500 psig, 9000 $mlg_{cat}^{-1}h^{-1}$, H_2:CO_2 ratio 3:1, In_2O_3:HZSM-5 mass ratio 1:1. The combined STY of CH_3OH and HC during the tandem reaction was ~3, ~10, and ~5 times higher over micro_In_2O_3/HZSM-5 as compared to In_2O_3||HZSM-5 at reaction temperatures of 350, 400, and 450°C, respectively.

S3.2. Effect of side reactions

During tandem CO_2 hydrogenation, MTH conversion could be facilitated at higher reaction temperatures aiding CH_3OH consumption,[4] however, the CH_3OH formation rates tend to drop as equilibrium favors the RWGS reaction.[5, 6] We observed this effect by increasing the reaction temperature from 350 to 450 °C, which increased CO selectivity from 77% to 93% while dropping CH_3OH selectivity from 5% to 1%, and C_{5+} selectivity from 51% to 7% (see **Figure S4A**). At the same time, C_2-C_4 selectivity increased from 38% to 58% by secondary hydrogenation driven by the high reaction temperature.[7] Therefore, higher reaction temperatures could inhibit the CH_3OH, olefin, and C_{5+} formation by favoring RWGS and secondary hydrogenation (illustrated in the inset of **Figure S4A**). We further probed the influence of secondary hydrogenation by increasing the H_2:CO_2 ratio in the feed from 1:1 to 4:1, which resulted in the C_2-C_4 paraffin selectivity increasing from 36% to 44% as shown in **Figure S4B**.

S3.3. Effect of CH_3OH synthesis step

We assessed whether higher CH_3OH synthesis rates could further enhance C_{5+} selectivity, the total reaction pressure was increased from 300 to 500 psig (350 °C, CO_2:H_2 ratio 3:1, and GHSV of 9000 $mlg_{cat}^{-1}h^{-1}$) as this facilitates CH_3OH synthesis.[8] We observed that the C_{5+} selectivity increased from 37% to 52% while CO_2 conversion increased from 24% to 29%. (see **Figure S4C**), due to enhanced CH_3OH formation with increasing pressures. We then modulated the CH_3OH synthesis rates by increasing the mass ratio of In_2O_3:HZSM-5 from 1:3 to 3:1 and observed an increase in CO_2 conversion from 19 to 31% along with an increase in C_{5+} selectivity from 46 to 55% (**Figure S4D**). Based on these observations we conclude that the presence of more CH_3OH could increase C-C coupling over acid sites of the HZSM-5 promoting methylation and higher C_{5+} selectivity.

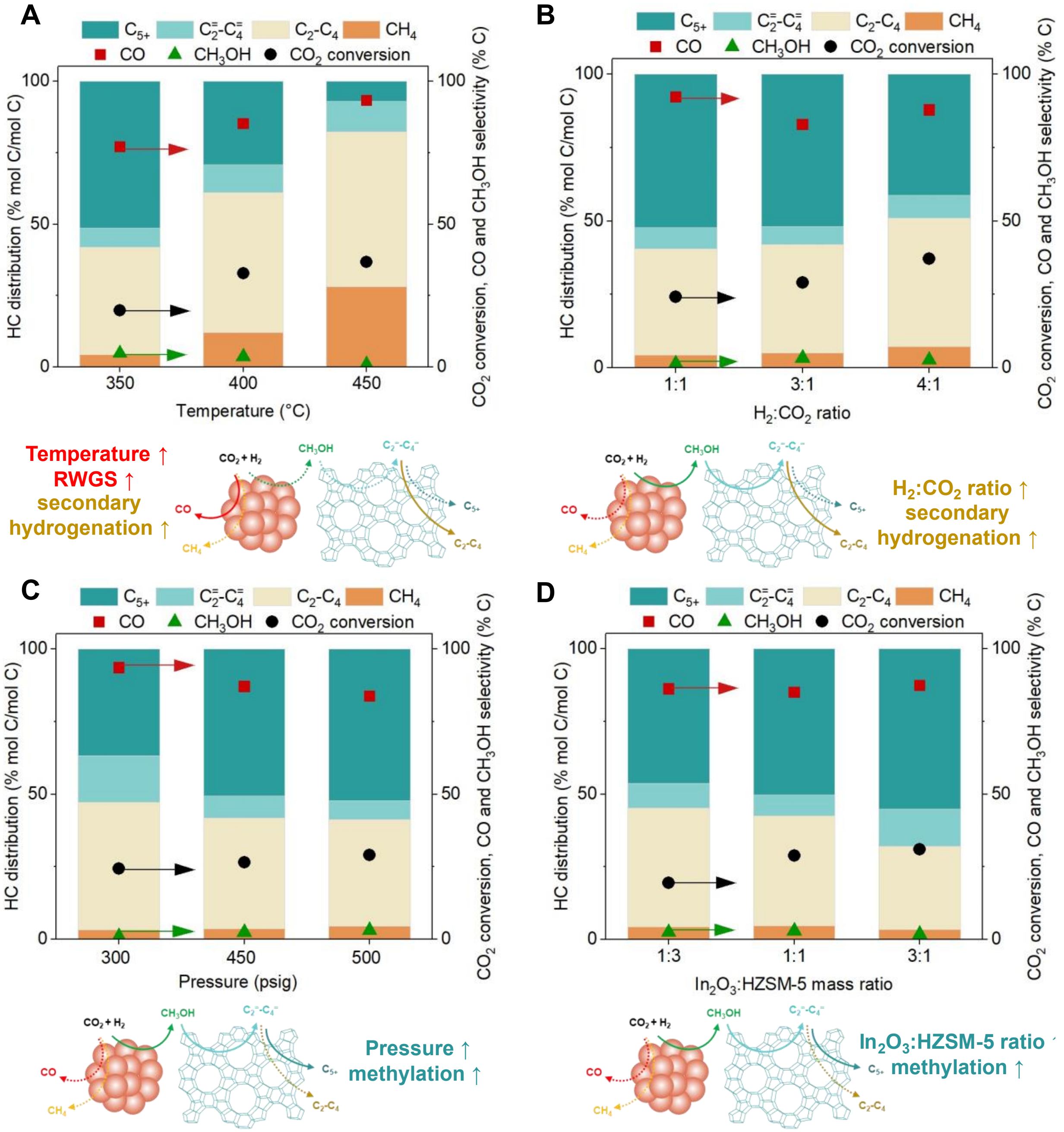

Figure S4: The influence of reaction pathways on the synergistic catalytic performance of micro_In_2O_3/HZSM-5 admixture, probed by modulating reaction conditions. A) Modulation of reaction temperatures. Reaction conditions: 500 psig, 9000 $mlg_{cat}^{-1}h^{-1}$, H_2:CO_2 ratio 3:1, In_2O_3:HZSM-5 ratio 1:1; B) Modulation of H_2:CO_2 ratios. Reaction conditions: 350 °C, 500 psig, 9000 $mlg_{cat}^{-1}h^{-1}$, In_2O_3:HZSM-5 ratio 1:1; C) Modulation of total pressures. Reaction conditions: 350 °C, 9000 $mlg_{cat}^{-1}h^{-1}$, H_2:CO_2 ratio 3:1,

In_2O_3:HZSM-5 ratio 1:1; D) Modulation of In_2O_3:HZSM-5 mass ratio. Reaction conditions: 350 °C, 500 psig, 9000 $mlg_{cat}^{-1}h^{-1}$, H_2:CO_2 ratio 3:1.

S3.4. Enhancing C-C coupling by increasing CH_3OH transfer via modulating GHSV

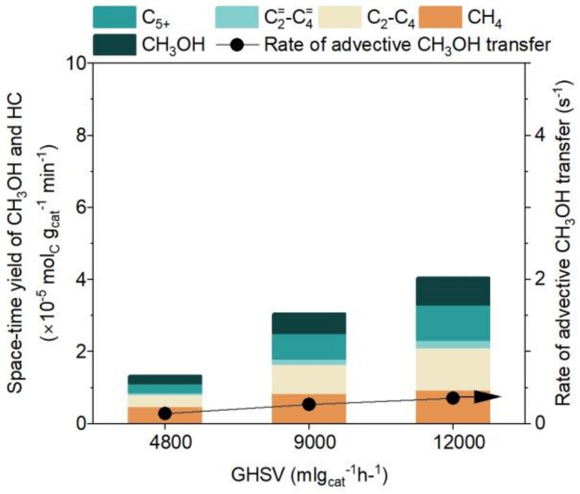

Figure S5: Effect of the transfer of CH_3OH intermediate from the redox sites of In_2O_3 to acid sites of HZSM-5 on catalytic performance over In_2O_3||HZSM-5. The figure depicts the space-time yield (STY) of HC and CH_3OH at different GHSV over In_2O_3||HZSM-5; Reaction conditions: 350 °C, 500 psig, H_2:CO_2 ratio 3:1, In_2O_3:HZSM-5 mass ratio 1:1. Enhancements in C_{2+} HC STY were observed by increasing GHSV and rate of CH_3OH advection.

S3.5. Stability and optimization of bifunctional admixtures

S3.5.1. micro_In_2O_3/HZSM-5

MTH conversion is known to be susceptible to zeolite deactivation *via* coke formation and blocking of the micropores by HCs.[9, 10] As micro_In_2O_3/HZSM-5 exhibited the highest space-time yields and C-C coupling selectivity, we evaluated its stability for tandem CO_2 hydrogenation for over 50 h at 350 °C (refer to **Figure S6A**). The HC distribution was found to be consistent over the reaction period with 5% CH_4, 40% C_2-C_4, 6% $C_2^=$-$C_4^=$, and 49% C_{5+} HCs. Furthermore, CO_2 conversion (22%), CO (78%), and CH_3OH (4.5%) selectivity were also found to be unaltered (see **Figure S6B**) indicating no deactivation of the microscale admixture over the reaction time up to 50 h, possibly due to the presence of H_2 inhibiting coke formation.[11]

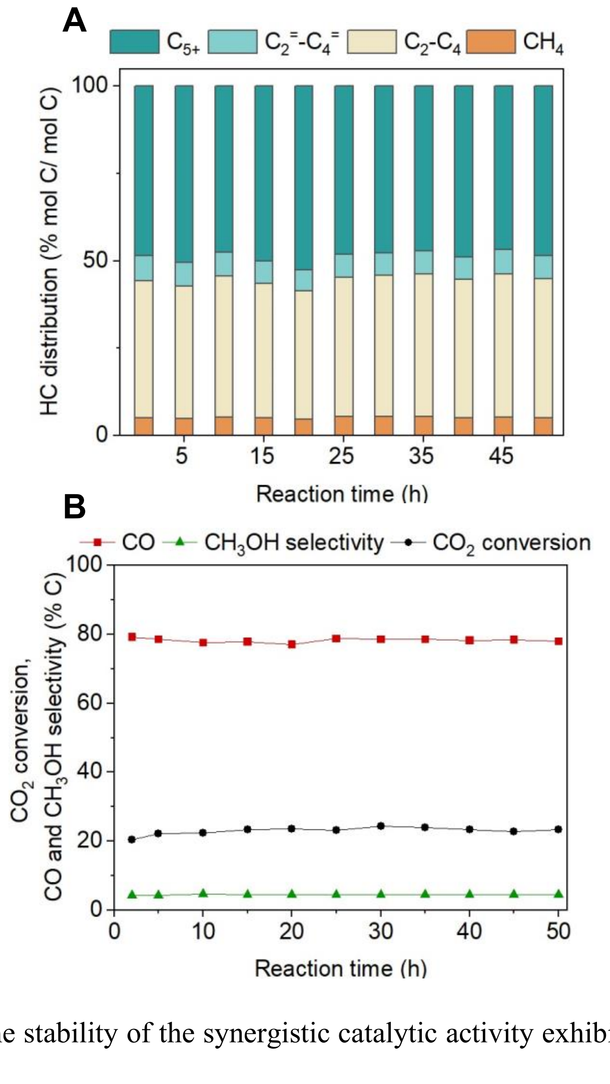

Figure S6: Analysis of the stability of the synergistic catalytic activity exhibited by micro_In_2O_3/HZSM-5. A) Hydrocarbon (HC) distribution B) CO_2 conversion, CO, and CH_3OH selectivity over

micro_In_2O_3/HZSM-5 catalyst. Reaction conditions: 350 °C, 500 psig, 9000 $mlg_{cat}^{-1}h^{-1}$, H_2:CO_2 ratio 3:1, In_2O_3:ZSM-5 mass ratio 1:1.

S3.5.2. nano_In_2O_3/HZSM-5

In the case of nano_In_2O_3/HZSM-5, only CH_4 forms as ion exchange occurs between Brønsted acid sites (BAS) and $In^{\delta+}$ species during the preparation process as discussed in the experimental section of the main text.

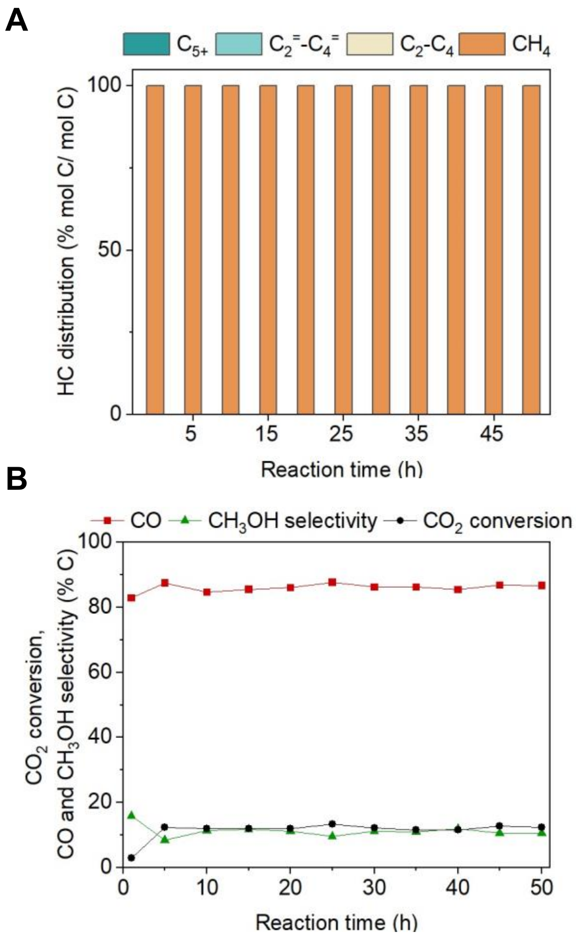

Figure S7: Analysis of the stability of the synergistic catalytic activity exhibited by nano_In_2O_3/HZSM-5. A) Hydrocarbon (HC) distribution B) CO_2 conversion, CO, and CH_3OH selectivity over nano_In_2O_3/HZSM-5 catalyst. Reaction conditions: 350 °C, 500 psig, 9000 $mlg_{cat}^{-1}h^{-1}$, H_2:CO_2 ratio 3:1, In_2O_3:ZSM-5 mass ratio 1:1.

S4. Theoretical studies

S4.1. Rate of CH_3OH advection

The time scale for advection, denoted as T_A, is approximately equal to the ratio of the characteristic length scale, L, to the flow velocity,[12, 13] v:

Advection Time Scale ≈ $T_A \sim L/v$

Rate of advection ≈ $1/T_A \sim v/L$

Velocity was calculated from flow rate and reactor tube diameter (0.77 cm) using ideal gas law. We assumed the velocity of CH_3OH molecules to be similar to the velocity of feed gas molecules.

Table S2: Rate of CH_3OH advection under reaction conditions of 350 °C, 500 psig, and H_2:CO_2 ratio 3:1 over In_2O_3||HZSM-5, micro_In_2O_3/HZSM-5, nano_In_2O_3/HZSM-5 admixtures. Characteristic length scales of the distance between the redox and acid sites in In_2O_3||HZSM-5, micro_In_2O_3/HZSM-5 nano_In_2O_3/HZSM-5 are ~10 mm and ~300 microns, and ~300 nm (estimated computationally in SI **section S1**), respectively.

GHSV ($mlg_{cat}^{-1}h^{-1}$)	Flowrate (ml/min)	Rate of CH_3OH advection (s^{-1})		
		**In_2O_3||HZSM-5**	**micro_In_2O_3/HZSM-5**	**nano_In_2O_3/HZSM-5**
4800	80	0.14	1.43	1430.68
9000	150	0.26	2.68	2682.53
12000	200	0.35	3.57	3576.71

S4.2. Equilibrium conversion of CO_2 during CH_3OH synthesis

The equilibrium CO_2 conversion and CH_3OH selectivity in CO_2 to CH_3OH reaction is shown in Figure S8.

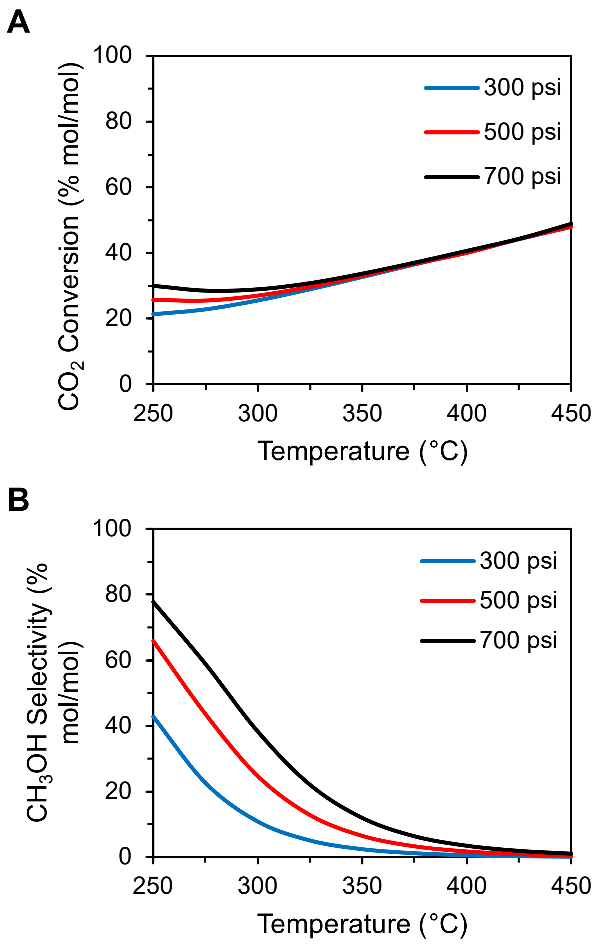

Figure S8: Equilibrium (A) CO_2 conversion and (B) CH_3OH selectivity in methanol synthesis at varying temperatures and pressures.

Equilibrium conversion and selectivity were calculated through the minimization of Gibbs free energy, G, using Microsoft Excel's solver function. The Gibbs free energy was represented as:

$$\frac{nG}{RT} = \sum n_i \left[\frac{\Delta_f G^o_{i,T}}{RT} + \ln(y_i P)\right] \tag{S11}$$

where n_i is the number of moles of component i, $\Delta_f G^o_{i,T}$ is the Gibbs free energy of formation of component i at temperature T, R is the universal gas constant, y_i is the mole fraction of component i, and P is the pressure.

The temperature dependent Gibbs free energy was derived from the following equations:

$$\frac{d(\frac{\Delta_f G_i^o}{T})}{dT} = \frac{\Delta_f H_i^o}{T^2} \tag{S12}$$

$$\Delta_f H_{i,T}^o = \Delta_f H_{i,T_R}^o + \int_{T_R}^{T} \Delta C_{p,i} dT \tag{S13}$$

$$\frac{C_{p,i}}{R} = A_i + B_i T + C_i T^2 + \frac{D_i}{T^2} \tag{S14}$$

where $\Delta_f H_{i,T}^o$ is the enthalpy of formation of component i at temperature T, $\Delta_f H_{i,T_R}^o$ is the enthalpy of formation of component i at a reference temperature T_R, and $C_{p,i}$ is the temperature dependent heat capacity of component i. The values of A, B, C, and D were obtained from a chemical engineering textbook.[14]

CO_2 conversion and CH_3OH selectivity were computed as follows:

$$CO_2 \text{ Conversion} = \frac{n_{CO_2,in} - n_{CO_2,out}}{n_{CO_2,in}} \times 100\% \tag{S15}$$

$$CH_3OH \text{ Selectivity} = \frac{n_{CH_3OH,out}}{n_{CH_3OH,out} + n_{CO,out}} \times 100\% \tag{S16}$$

where n is the number of moles.

S4.3. Density functional theory (DFT) calculations

Spin-polarized density functional theory (DFT) calculations were performed using the Vienna *Ab initio* Simulation Package (VASP) code, version 5.4.4.[15] We used a plane wave basis set with kinetic energy expansion cut off energy of 450 eV (1 eV = 96.5 kJ/mol). The generalized gradient approximation of Perdew-Burke-Ernzerhof (PBE) was employed to capture the electronic exchange and correlation interactions.[16] The Projector Augmented Wave (PAW) method was used to treat the core- valence electron interactions for all the optimizations. The DFT calculations do not account for the dispersion interactions intrinsically.[17] Hence, these were incorporated using Grimme's DFT-D3 method implemented in VASP.[18] The geometries were fully relaxed until the residual forces were smaller than 0.1 eV/ Å, and the self-consistent energy convergence was set to 1.0×10^{-5} eV/atom.

The MFI structure for HZSM-5 that we modelled was composed of 96 Si and 192 O atoms. The optimized lattice parameters of a = 20.25 Å, b = 19.89 Å, and c = 13.24 Å agreed with X-ray Diffraction (XRD) findings in the literature. [19] BAS were created by the isomorphic substitution of two Si atoms with Al atoms at the T7 and T11 sites of ZSM-5, satisfying the Lowenstein's rule, and incorporating two protons for charge compensation to form HZSM-5. This represents an Si:Al ratio of 47 which mimics the Si:Al ratio of 40 in the catalyst used in the experiments. The Brillouin zone sampling of the HZSM-5 structure was restricted to the gamma point. To model the ion exchanged In complex in ZSM5, an In_2O moiety which is commonly documented in the literature was considered.[20] We incorporated the In_2O complex in the ZSM5 model by replacing the protons. This represented the ion-exchanged extra framework moiety over the Al sites of ZSM-5. This catalyst model is hereafter referred to as In_2O/ZSM-5 and is shown in **Figure S9**. The computed In-O-In stretching band at 361 cm^{-1} correlates with the In-O-In stretch at 382 cm^{-1} identified during Raman analysis.

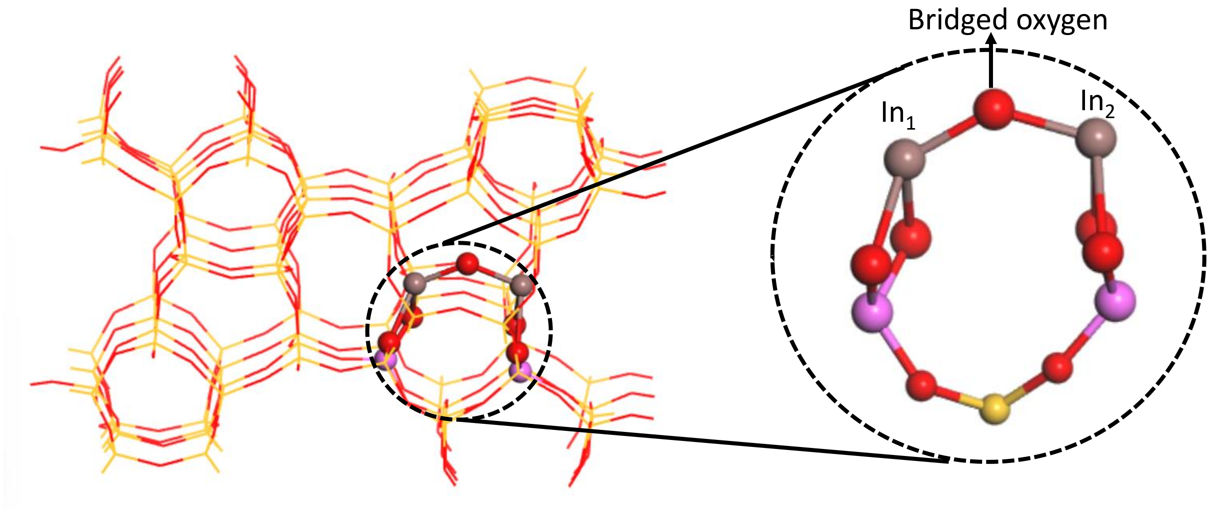

Figure S9: In_2O/ZSM5 catalyst structure with In_2O complex (circled and zoomed out) incorporated as an extra framework moiety over the Al sites of ZSM5. Color code of the atoms: Red - O; Magenta – Al; Orange-Yellow - Si; Greyish mauve -In.

Methanol hydrogenolysis reaction mechanisms were investigated using the In_2O/ZSM-5 catalyst. The Nudged Elastic Band (NEB) method was used to estimate the minimum energy paths and the respective transition states for each of the elementary steps. The identified transition states were further refined using the Improved Dimer Method (IDM) implemented in VASP.[21] Stationary points corresponding to minima and first order saddle points on the potential energy surface were ascertained based on harmonic vibrational frequency analysis. The following equations were then used to calculate the corresponding adsorption energies and the activation energy barriers.

The adsorption energy of a species was calculated using the formula:

$$E_{ads} = E_{slab + species} - E_{slab} - E_{gas\ species} \quad (S17)$$

Where E_{ads} is the adsorption energy of the species, $E_{slab + species}$ is the energy of the system where species is adsorbed on the catalyst slab, E_{slab} is the energy of the pure catalyst slab, and $E_{gas\ species}$ is the energy of the species in the gas phase.

The activation energy barrier was calculated as follows:

$$E_a = E_{TS} - E_r \quad (S18)$$

Where E_a is the activation energy barrier of the elementary reaction steps, E_{TS} is the energy of the transition state complex, and E_r is the energy of the reactant intermediate in eV.

The free energy profile for the investigated reaction network was obtained by correcting the DFT derived ground state energies with the Zero Point Vibrational Energies (ZPVE) and the enthalpic and entropic contributions at 350°C, using standard statistical mechanics relationships.

S5. Characterization

S5.1. Structural, textural, and morphological characterizations of In_2O_3 and HZSM-5

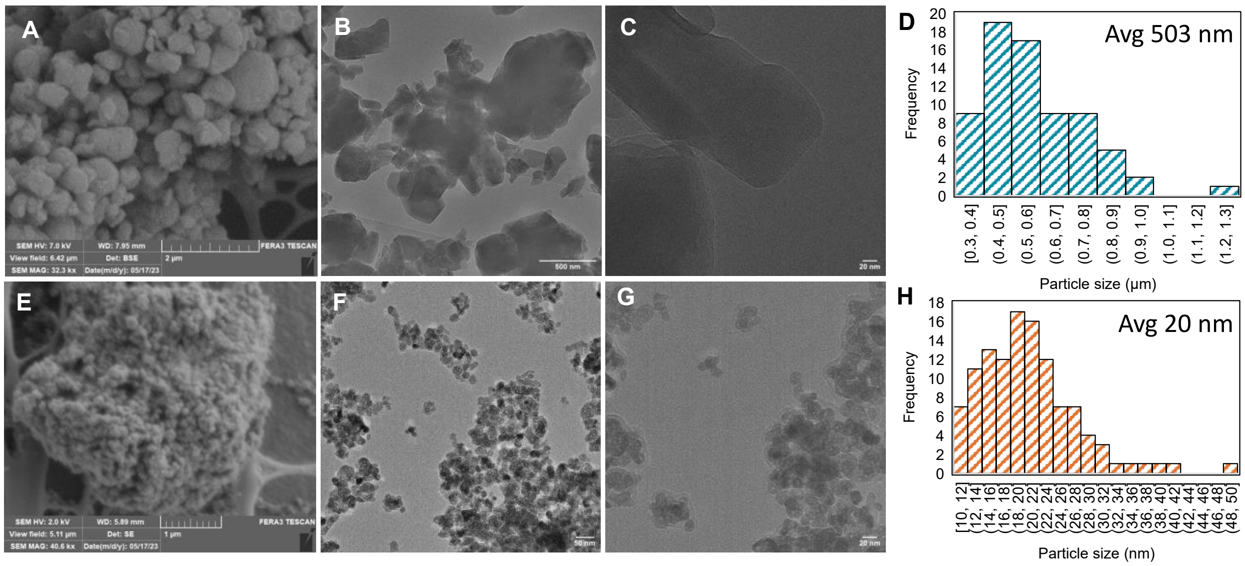

Figure S10: Assessment of structural and morphological properties of HZSM-5 and In_2O_3. (A) Scanning electron microscopy (SEM) of pristine HZSM-5; B) Transmission electron microscopy (TEM) of pristine HZSM-5 at 25000× magnification; C) TEM of HZSM-5 at 50000× magnification; D) Particle size distribution of HZSM-5 showing average particle size of 503 nm; E) SEM of In_2O_3; F) TEM of In_2O_3 at 29000× magnification; G) TEM of In_2O_3 at 50000× magnification; H) Particle size distribution of In_2O_3 showing average particle size of 20 nm, computed manually.

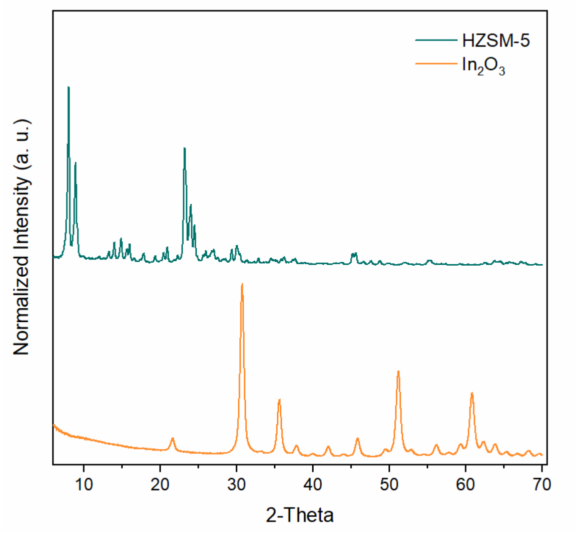

Figure S11: A) Powder X-ray Diffraction (PXRD) pattern of HZSM-5 (green) exhibiting characteristic peaks at 7.9° (101), 8.9° (200), 23.0° (501), 23.6° (341), and 24.4° (303). PXRD pattern for HZSM-5 was normalized with respect to the peak intensity at 7.9°; B) PXRD pattern of In_2O_3 (orange) showing characteristic peaks of cubic In_2O_3 at 30.8° (222), 35.5° (400), 51.4° (440), and 60.7° (622). PXRD pattern was normalized with respect to the In_2O_3 peak at 30.8°.

S5.2. Structural, textural, and morphological characterizations of nano_In_2O_3/HZSM-5

Powder X-ray diffraction (PXRD) patterns of In_2O_3, HZSM-5, micro_In_2O_3/HZSM-5, and nano_In_2O_3/HZSM-5 are shown in **Figure S12**. The characteristic peaks of cubic In_2O_3 at 30.8° (222), 35.5° (400), 51.4° (440), and 60.7° (622), and HZSM-5 at 7.9° (101), 8.9° (200), 23.0° (501), 23.6° (341), and 24.4° (303) were seen for both micro_In_2O_3/HZSM-5 and nano_In_2O_3/HZSM-5.[22-26] No peaks were observed for metallic In (at 32.9°, 36.5°, and 39.2°),[27, 28] or $In(OH)_3$ (at 22.26°, 51.16°, and 56.46°).[29] However, for nano_In_2O_3/HZSM-5, the relative intensity of peaks corresponding to HZSM-5 decreased relative to peaks corresponding to In_2O_3 (as compared to micro_In_2O_3/HZSM-5 at the same mass ratio of In_2O_3:HZSM-5), suggesting that the powder mixing for the synthesis of nano_In_2O_3/HZSM-5 may cause some structural damage to HZSM-5, as suggested by previous reports.[30] Though, a comparison of scanning electron micrographs (SEM) (**Figure S10A**), and transmission electron micrographs (TEM) of HZSM-5 (**Figure S10B, S10C**), and nano_ In_2O_3/HZSM-5, as shown in **Figure S12B, S12C**, revealed similar average particle size for HZSM-5 (~500 nm) and no structural damage to HZSM-5 during the mixing process (to prepare nano_In_2O_3/HZSM-5).

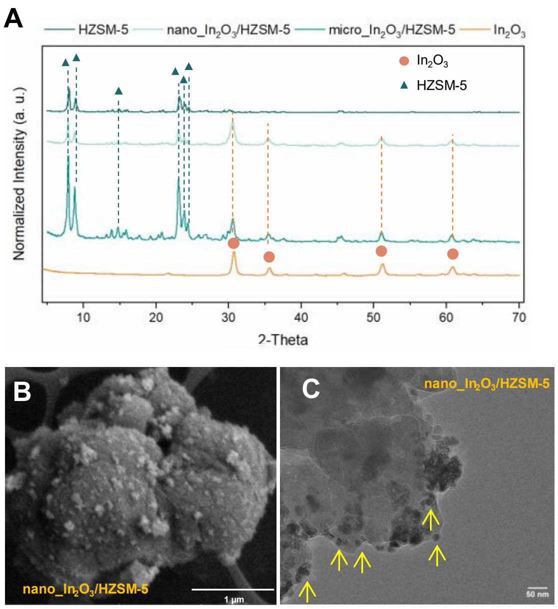

Figure S12: Assessment of structural and morphological properties of nano_In_2O_3/HZSM-5. A) Powder X-ray diffraction (PXRD) patterns of In_2O_3, HZSM-5, micro_In_2O_3/HZSM-5, and nano_In_2O_3/HZSM-5. B) Scanning electron microscopy (SEM) of nano_In_2O_3/HZSM-5. C) Transmission electron microscopy (TEM) of nano_In_2O_3/HZSM-5. Yellow arrows point to In_xO_y clusters on the surface of HZSM-5.

S5.3. Physisorption analysis of bifunctional admixtures

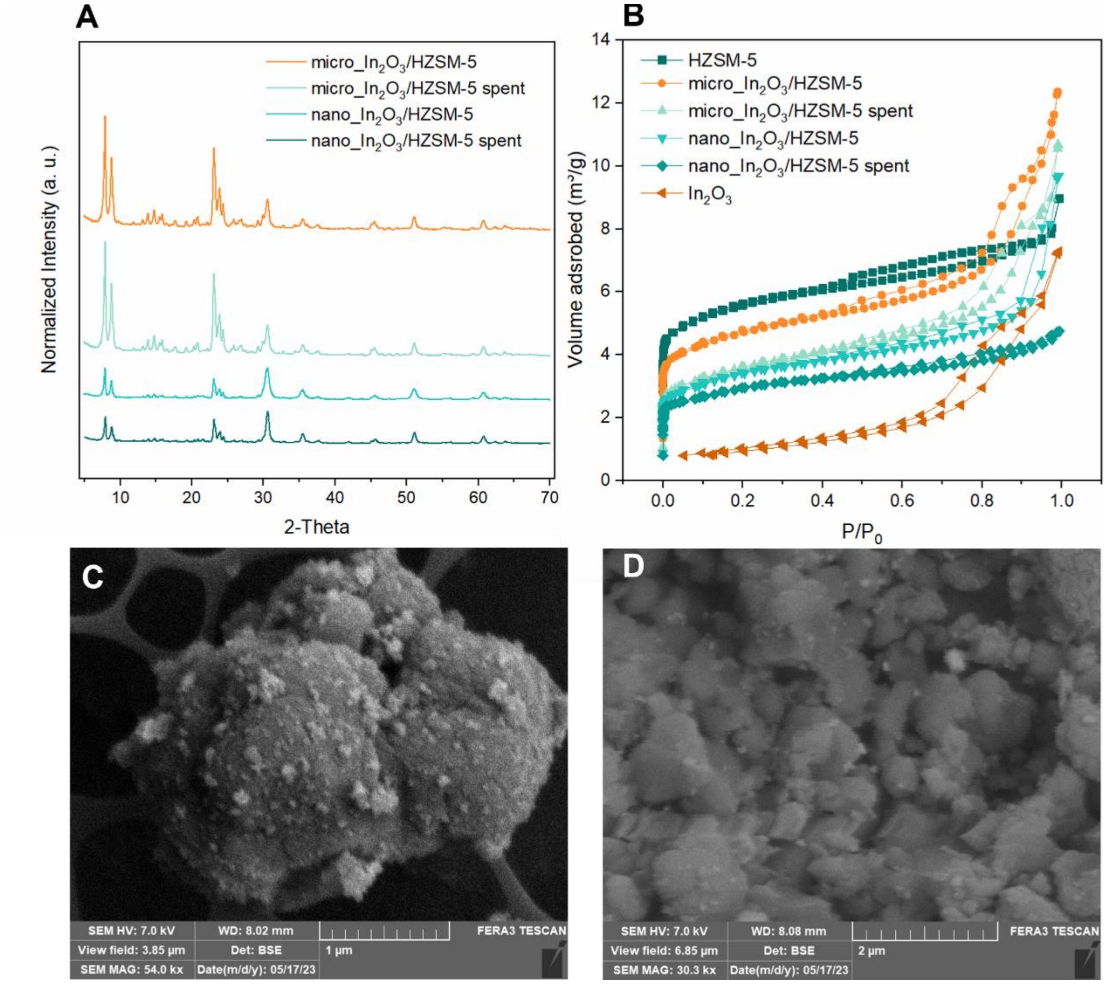

Figure S13: A) Powder X-ray Diffraction (PXRD) pattern of fresh and spent micro_In_2O_3/HZSM-5 and nano_In_2O_3/HZSM-5 catalyst. The characteristic peaks of cubic In_2O_3 at 30.8° (222), 35.5° (400), 51.4° (440), and 60.7° (622), and HZSM-5 at 7.9° (101), 8.9° (200), 23.0° (501), 23.6° (341), and 24.4° (303) were seen for both fresh and spent catalysts of micro_In_2O_3/HZSM-5 and nano_In_2O_3/HZSM-5 admixtures. However, the relative intensity of peaks corresponding to HZSM-5 decreased in fresh and spent nano_In_2O_3/HZSM-5 relative to peaks corresponding to In_2O_3 (as compared to micro_In_2O_3/HZSM-5 at the same mass ratio of In_2O_3:HZSM-5); PXRD patterns of micro_In_2O_3/HZSM-5 and nano_In_2O_3/HZSM-5 were normalized with respect to the In_2O_3 peak at 30.8°. B) N_2 adsorption isotherm of In_2O_3, HZSM-5 and

fresh and spent micro_In_2O_3/HZSM-5 and nano_In_2O_3/HZSM-5 catalyst. HZSM-5 exhibits type I isotherm for microporous structure, while In_2O_3 exhibits mesoporous type IV isotherm. Both micro_In_2O_3/HZSM-5 and nano_In_2O_3/HZSM-5 admixtures exhibit mesoporous type IV isotherm having In_2O_3 in the admixture; C, D) SEM imaging of fresh nano_In_2O_3/HZSM-5 showing HZSM-5 particle shape and structure retained in the admixture.

Table S3: Brunauer-Emmett-Teller (BET) surface area and pore volume measurements for fresh and spent In_2O_3/HZSM-5 bifunctional catalysts from N_2 physisorption.

		**BET surface area**	**slope**	**BET int**	**Corr. Coeff. BET**	**pore vol**	**t-plot**	**micropore vol**	**Micropore area**	**external surface area**	**Correlation coeff**
**Sample**	P/P_o	m^2/g	1/g	1/g		cc/g	P/P_o	cc/g	m^2/g	m^2/g	
**In_2O_3**	0.1-0.3	47.52	72.63	0.65	1.00	0.16	0.2-0.5	0.00	0.00	0.00	
**micro_In_2O_3/HZSM-5**	0.01-0.1	256.00	13.59	0.01	1.00	0.29	0.2-0.5	0.07	171.98	84.02	1.00
**spent micro_In_2O_3/HZSM-5**	0.01-0.1	227.16	15.33	0.01	1.00	0.16	0.2-0.5	0.07	163.82	63.35	0.99
**nano_In_2O_3/HZSM-5**	0.01-0.1	201.83	17.24	0.02	1.00	0.25	0.2-0.5	0.06	129.95	71.87	1.00
**spent nano_In_2O_3/HZSM-5**	0.01-0.1	193.99	17.93	0.02	1.00	0.25	0.2-0.5	0.05	112.08	81.91	1.00
**HZSM-5**	.005-.08	513.85	6.77	0.00	1.00	0.35	0.2-0.5	0.17	392.07	121.77	1.00

S5.4. Structural, morphological, and spectroscopic characterization of xInZSM-5

PXRD patterns of xInZSM-5 (see **Figure S14A**) were compared with nano_In_2O_3/HZSM-5. None of the samples exhibited peaks for metallic In (at 32.9°, 36.5°, and 39.2°),[27, 28] or $In(OH)_3$ (at 22.26°, 51.16°, and 56.46°).[29] Further, PXRD patterns of 0.3InZSM-5 and 0.7InZSM-5 did not exhibit any characteristic peaks of In_2O_3, likely due to In_2O_3 crystallites being beyond the detection limits of PXRD (<4 nm) and/or ion exchange of BAS with $In^{\delta+}$ at the low In:Al loadings.[22, 23] This is further evident from SEM (**Figure S14B, S14C**) and transmission electron microscopy (TEM) (**Figure S14D, S14E)** micrographs of 0.3InZSM-5 and 0.7InZSM-5**,** where very few In_xO_y clusters (~10 nm size, indicated by yellow arrows) were visible on the HZSM-5 surface.[20, 31] Additionally, some smaller particles of ~3 nm were seen in the TEM micrographs (zoomed-in images are shown in **Figures S15, S16**), which may be related to $In^{\delta+}$ ion-exchanged species *(vide infra)*. For 3.5InZSM-5, the characteristic peaks of In_2O_3 were visible in PXRD in **Figure 14A**, consistent with SEM and TEM micrographs (shown in **Figure 14E, 14F)**, where larger clusters of In_xO_y (~20 nm) were found on the HZMS-5 surface. Again, a few smaller particles of ~3 nm were visible in TEM (zoomed-in images are shown in **Figure S17**). Notably, the SEM (**Figure S13B)** and TEM (**Figure S13C)** micrographs of nano_In_2O_3/HZSM-5 showed similar sizes of In_xO_y clusters as 3.5InZSM-5 (**Figure S17**). Taken together, the PXRD, SEM, and TEM data suggest that nano_In_2O_3/HZSM-5 has similar structural and morphological properties as 3.5InZSM-5.[3, 32] However, the ion exchange between BAS and $In^{\delta+}$ could not be definitively probed from SEM and TEM micrographs.

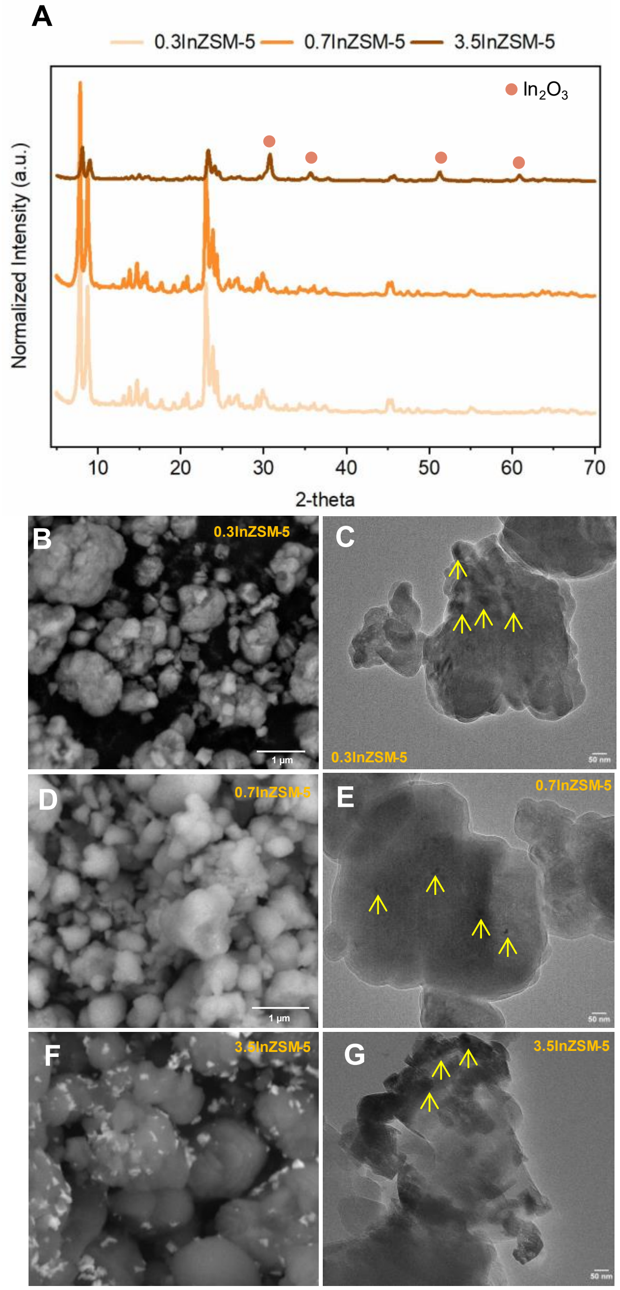

Figure S14: Structural, morphological, and spectroscopic characterization of $In^{\delta+}$ ion-exchanged HZSM-5 or xInZSM-5 (x = In:Al = 0.3, 0.7, 3.5). A) Powder X-ray diffraction (PXRD) pattern of 0.3InZSM-5, 0.7InZSM-5, and 3.5InZSM-5. (B) Scanning electron microscopy (SEM) of 0.3InZSM-5, D) 0.7InZSM-5, F) 3.5InZSM-5, and (C) Transmission electron microscopy (TEM) of 0.3InZSM-5, E)

0.7InZSM-5, G) 3.5InZSM-5 at 25000× magnification. Yellow arrows point to In_xO_y clusters on the surface of HZSM-5.

S5.5. TEM characterization of ion-exchanged xInZSM-5 and nano_In_2O_3/HZSM-5

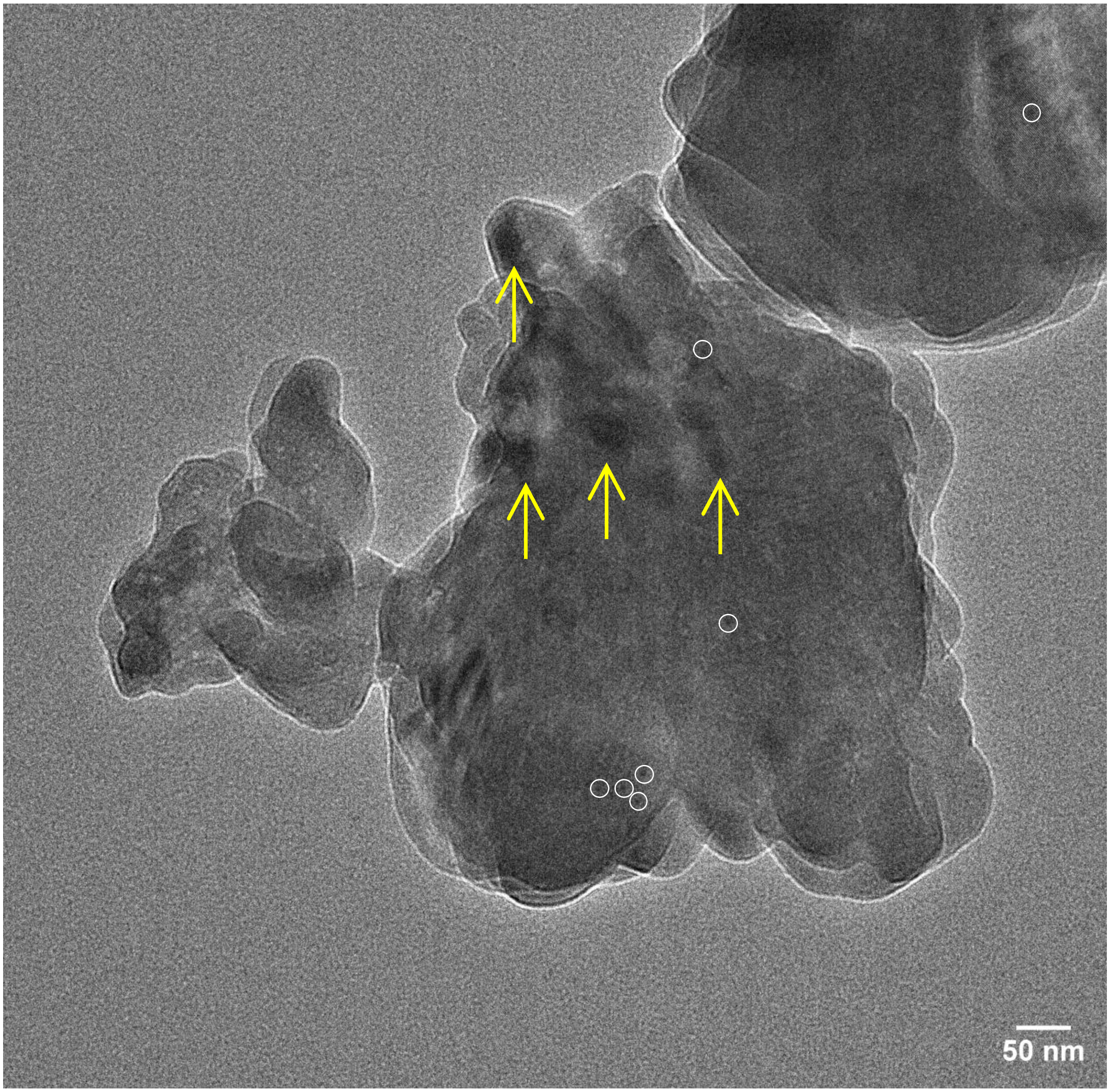

Figure S15: Transmission electron microscopy (TEM) of ion-exchanged 0.3InZSM-5 (In:Al ratio 0.3:1) at 25000× magnification. Yellow arrows point out ~10 nm In_xO_y clusters on the surface of HZSM-5 and white dashed circles show smaller ~3 nm particles, which may be related to $In^{\delta+}$ ion-exchanged species *(vide infra)*.

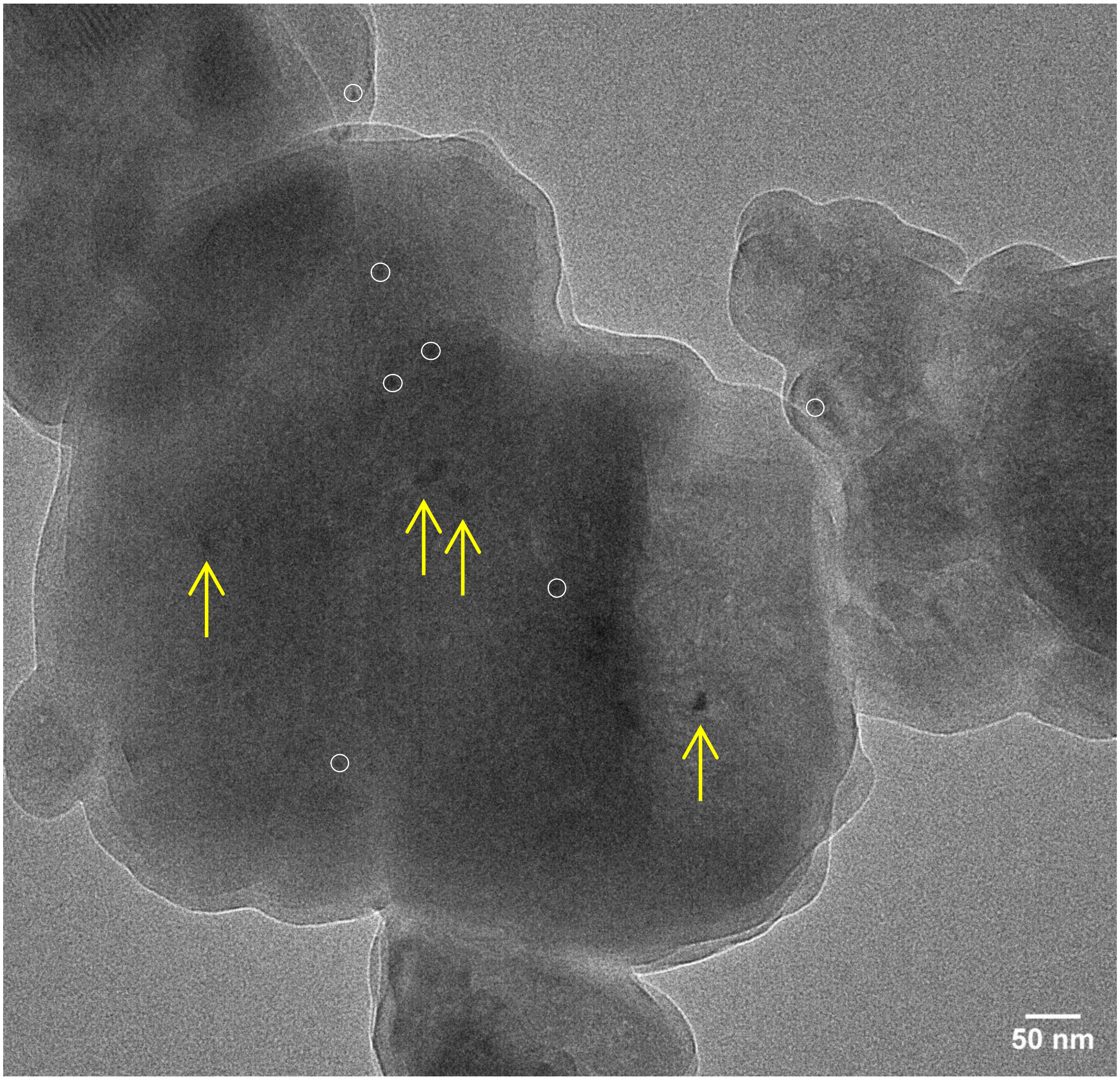

Figure S16: Transmission electron microscopy (TEM) of ion-exchanged 0.7InZSM-5 (In:Al ratio 0.7:1) at 25000× magnification. Yellow arrows point out ~10 nm In_xO_y clusters on the surface of HZSM-5 and white dashed circles show smaller ~3 nm particles, which may be related to $In^{\delta+}$ ion-exchanged species *(vide infra)*.

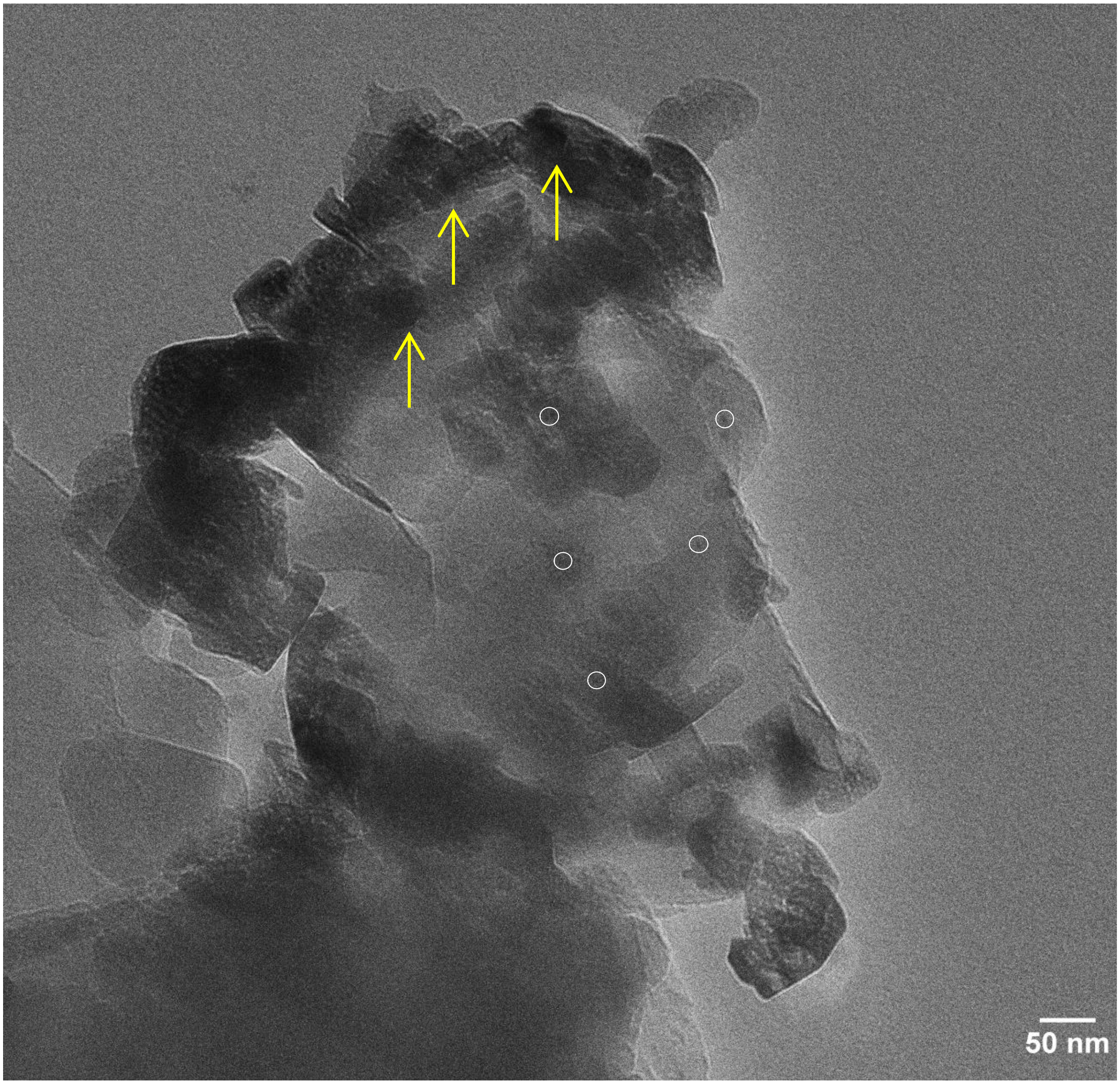

Figure S17: Transmission electron microscopy (TEM) of ion-exchanged 3.5InZSM-5 (In:Al ratio 3.5:1) at 25000× magnification. Yellow arrows point out ~20 nm In_xO_y clusters on the surface of HZSM-5 and white dashed circles show smaller ~3 nm particles, which may be related to $In^{\delta+}$ ion-exchanged species *(vide infra)*.

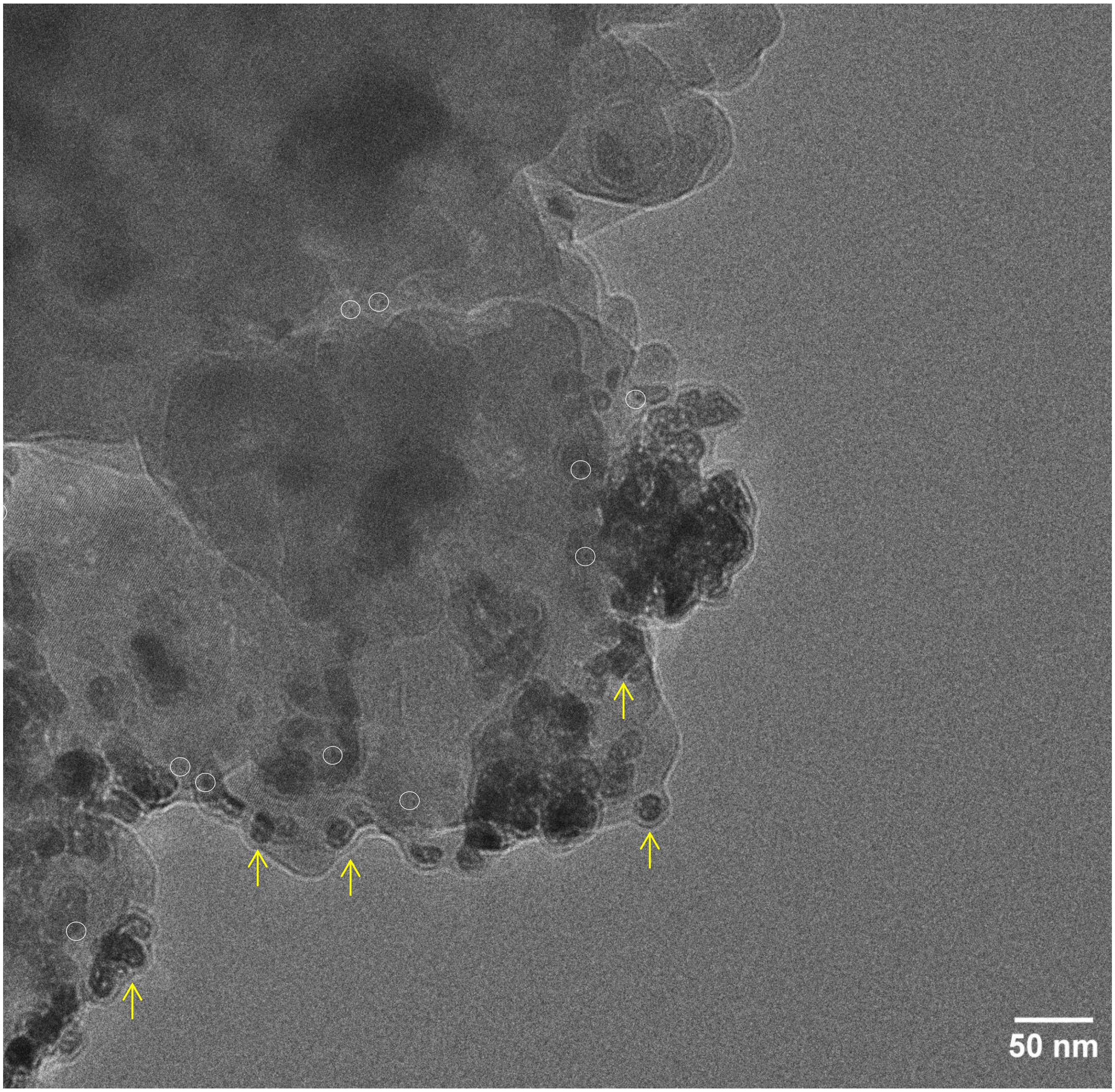

Figure S18: Transmission electron microscopy (TEM) of nano_In_2O_3/HZSM-5 (1:1) at 25000× magnification. Yellow arrows point out ~20 nm In_xO_y clusters on the surface of HZSM-5 and white dashed circles show smaller ~3 nm particles, which may be related to $In^{\delta+}$ ion-exchanged species *(vide infra)*.

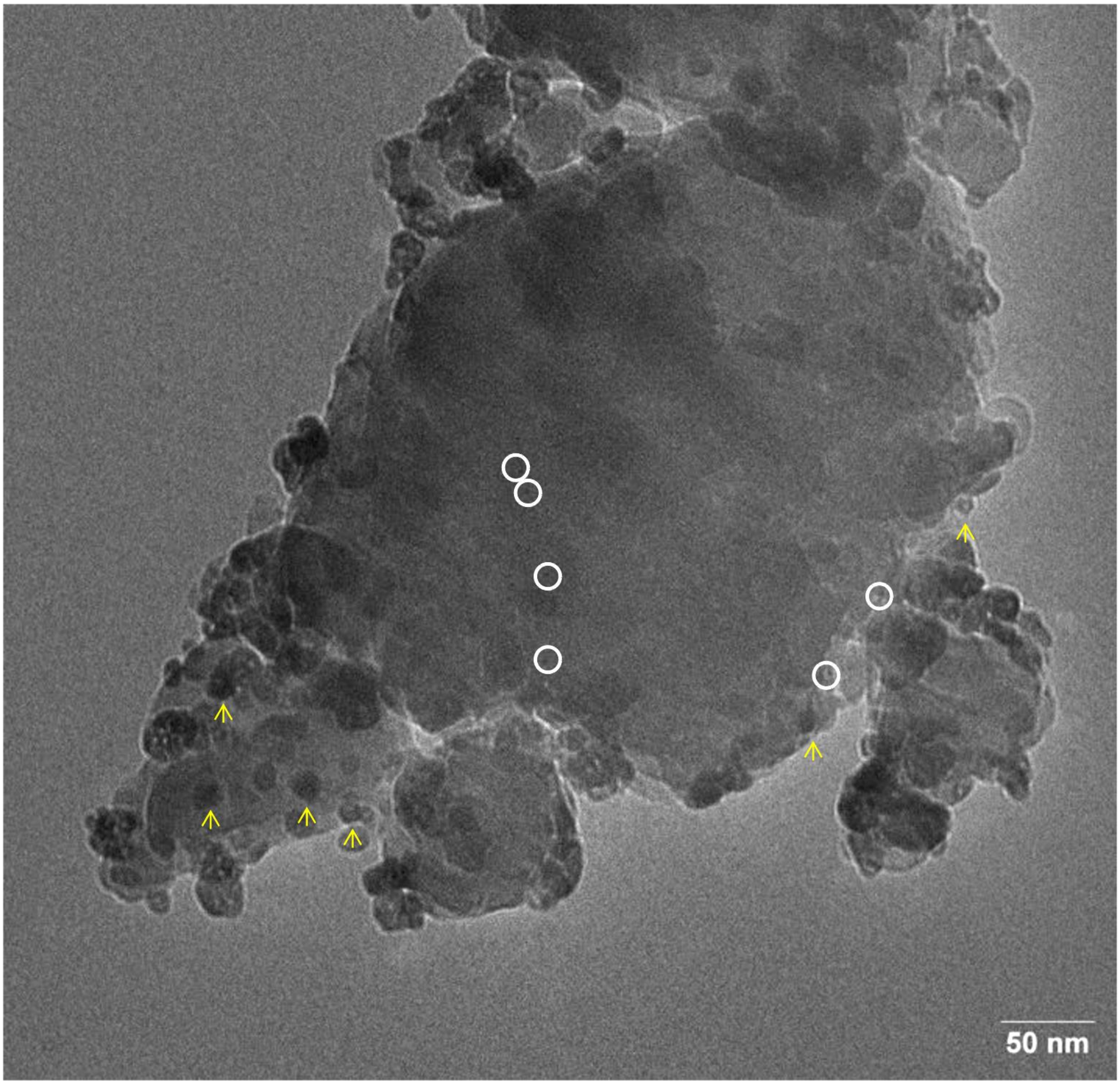

Figure S19: Transmission electron microscopy (TEM) of nano_In_2O_3/HZSM-5 (1:5) at 25000× magnification. Yellow arrows point out ~20 nm In_xO_y clusters on the surface of HZSM-5 and white dashed circles show smaller ~3 nm particles, which may be related to $In^{\delta+}$ ion-exchanged species *(vide infra)*.

S5.6. Analysis of Raman spectra

The ratio of bands at 370 (I_2) and 308 (I_1) cm^{-1} in Raman spectra of In_2O_3 indicates the extent of oxygen vacancies on In_2O_3.[33-36] **Figure S20B** shows that nano_In_2O_3/HZSM-5 (1:1) and nano_In_2O_3/HZSM-5 (1:5) have a higher ratio of I_2/I_1 (0.85 and 1.03, respectively) compared to pure In_2O_3 (0.34), indicating more oxygen vacancies in the admixtures compared to bulk In_2O_3. However, the shoulder at 382 cm^{-1} might not be related to the oxygen vacancies alone, as nano_In_2O_3/HZSM-5 (1:5) has a lesser amount of In_2O_3 in the admixture than nano_In_2O_3/HZSM-5 (1:1) but exhibits a higher ratio of I_2/I_1 (1.01) compared to nano_In_2O_3/HZSM-5 (1:1) (0.52). Therefore, we hypothesize the shoulder at 382 cm^{-1} could also be related to the ion exchange of $In^{\delta+}$ with H^+. By further analyzing the I_2/I_1 for ion-exchanged xInZSM-5 samples, we observed increased values (I_2/I_1 ratio 1.15, 1.16, and 1.17 for x=0.3, 0.7 and 3.5, respectively) compared to nano admixtures. Hence, the shifting of the band from 370 cm^{-1} to 382 cm^{-1} was likely related to the ion exchange of $In^{\delta+}$ with zeolitic H^+.

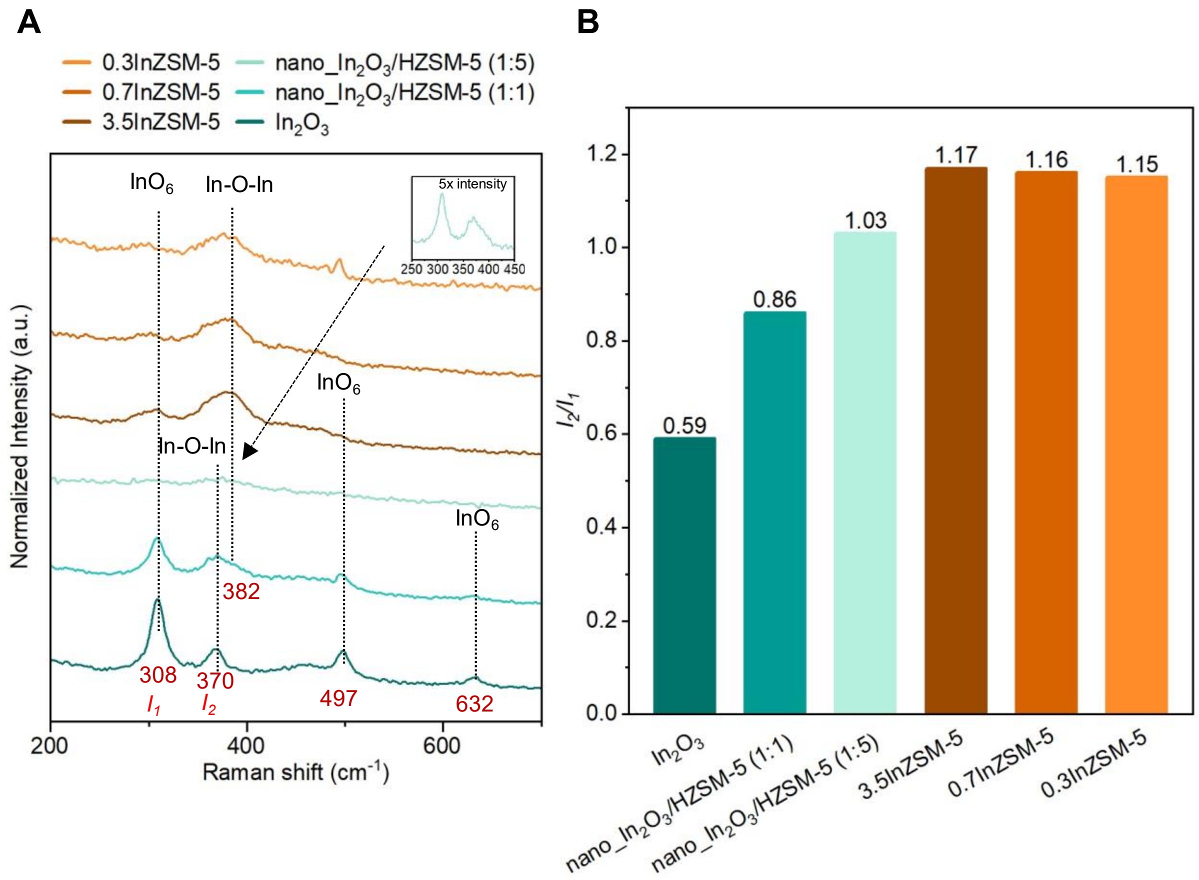

Figure S20: A) Raman spectra for In_2O_3, nano_In_2O_3/HZSM-5 (1:1), nano_In_2O_3/HZSM-5 (1:5), 0.3InZSM-5, 0.7InZSM-5, and 3.5InZSM-5. The inset on the top right shows zoomed-in spectra for nano_In_2O_3/HZSM-5 (1:5) at 5x intensity; B) I_2/I_1 ratio of In_2O_3, nano_In_2O_3/HZSM-5 (1:1), nano_In_2O_3/HZSM-5 (1:5), 3.5InZSM-5, and 0.7InZSM-5 catalysts, calculated from Raman spectra.

Table S4: Ratio of bands at 370 (I_2) and 303(I_1) cm^{-1} in Raman spectra

Sample	Ratio (I_2/I_1)
**In_2O_3**	0.59
**In_2O_3/HZSM-5_G**	0.57
**In_2O_3/HZSM-5_M (1:1)**	0.86
**In_2O_3/HZSM-5_M (1:5)**	1.03
**0.3In-ZSM-5**	1.15
**0.7In-ZSM-5**	1.16
**3.5In-ZSM-5**	1.17

S5.7. Parameters for X-ray photoelectron spectroscopy (XPS) analysis

Table S5: Assignments and constraints for XPS data analysis

Species	Label	Position (eV) Constraint	FWHM (eV) Constraint	Line Shape
**C-C, C-H**	A	284.8	0.7 to 1.5	GL(30)
**C-OH, C-O-C**	B	A+1.5	A*1	GL(30)
**C=O**	C	A+3.0	A*1	GL(30)
**O-C=O**	D	A+4.0	A*1	GL(30)

Table S6: Assignments and constraints for XPS data analysis for ion-exchanged In-ZSM-5

Species	Label	Position (eV) Constraint	FWHM (eV) Constraint	Peak Area Constraint	Line Shape
**In^{3+} $3d_{5/2}$**	A	443.9	NA	NA	GL(30)
**In^{3+} $3d_{3/2}$**	B	A+7.54	A*1	A*0.67	GL(30)
**In/ZSM-5 $3d_{5/2}$**	C	NA	NA	NA	GL(30)
**In/ZSM-5 $3d_{3/2}$**	D	C+7.54	C*1	C*0.67	GL(30)
**In^{+} $3d_{5/2}$**	E	NA	NA	NA	GL(30)
**In^{+} $3d_{3/2}$**	F	E+7.54	E*1	E*0.67	GL(30)

S5.8. XRD of 3.5InZSM-5 and nano_In_2O_3/HZSM-5 (1:5)

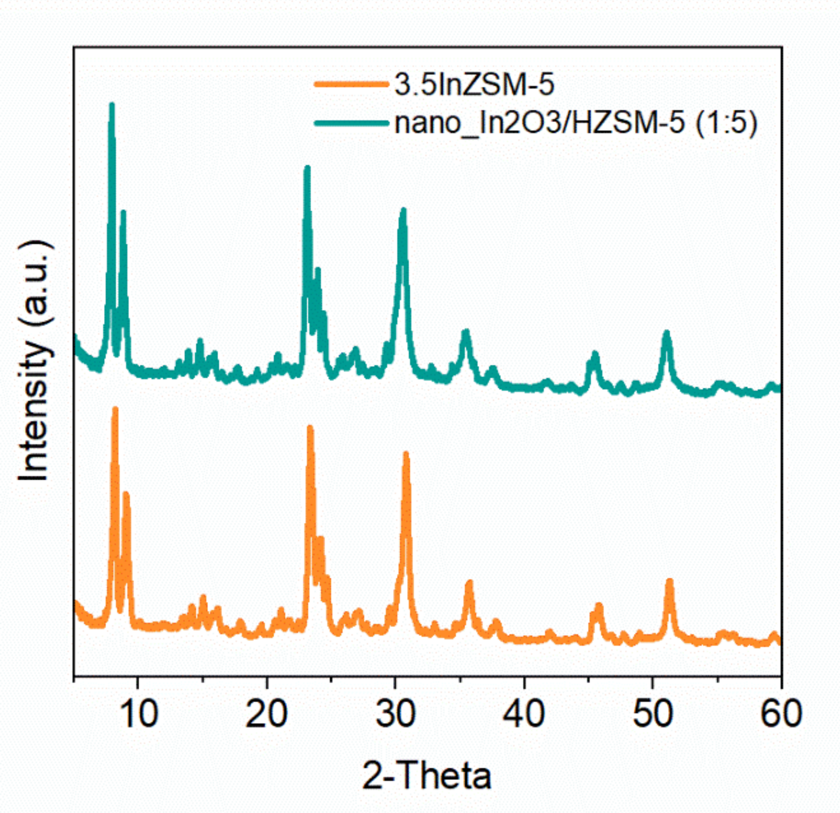

Figure S21: Powder X-ray Diffraction (PXRD) patterns of 3.5InZSM-5 and nano_In_2O_3/HZSM-5 (1:5) with equivalent In^{3+} loading. The characteristic peaks of cubic In_2O_3 at 30.8° (222), 35.5° (400), 51.4° (440), and 60.7° (622), and HZSM-5 at 7.9° (101), 8.9° (200), 23.0° (501), 23.6° (341), and 24.4° (303) were seen for both cases, indicating similar composition with In_xO_y particles on the surface. PXRD patterns were normalized with respect to the In_2O_3 peak at 30.8°.

S6. Carbon balance and reactivity data

Table S7: C balance during CO_2 hydrogenation reaction over micro_In_2O_3/HZSM-5 at different reaction temperatures. Reaction conditions: 500 psig, 9000 $mlg_{cat}^{-1}h^{-1}$, H_2:CO_2 ratio 3:1, In_2O_3:HZSM-5 mass ratio 1:1.

**T (°C)**	**CO_2 in (%)**	**C_{in} or $CO_{2,in}$ (×10^{-3} molC/min)**	**CO_2 out (C%)**	**$CO_{2,out}$ (×10^{-3} molC/min)**	**CO_2 conversion**	**sum of C in HC (C%)**	**sum of C in CO (C%)**	**C_{out} (HC+CO + unconverted CO_2) (×10^{-3} molC/min)**	**CO_2 conv. internal norm. (C%)**	**C balance (%)**
330	25.5	**1.71**	23.83	1.49	12.76	0.94	1.86	**1.67**	10.54	97.51
350	24.9	**1.75**	18.45	1.27	27.59	0.81	5.24	**1.69**	24.72	96.17
400	25.5	**1.71**	19.89	1.21	29.28	1.51	7.03	**1.73**	30.08	101.14
450	25.5	**1.71**	17.98	1.04	38.89	0.67	9.42	**1.63**	35.94	95.40

$$CO_2 \text{ conversion, } X_{CO_2} = \frac{C_{CO_2,\text{ inlet}} \cdot F_{\text{inlet}} - C_{CO_2,\text{ outlet}} \cdot F_{\text{outlet}}}{C_{CO_2,\text{ inlet}} \cdot F_{\text{inlet}}} \times 100\% \qquad \text{(S19)}$$

Internal normalization method,

$$CO_2 \text{conversion, } X_{CO_2,\text{internal normalization}} = \frac{RRF_{CH_3OH} \times A_{CH_3OH} + RRF_{CO} \times A_{CO} + \sum_1^n RRF_{C_nH_m} \times A_{C_nH_m}}{RRF_{CO_2,\text{outlet}} \times A_{CO_2,\text{outlet}} + RRF_{CH_3OH} \times A_{CH_3OH} + RRF_{CO} \times A_{CO} + \sum_1^n RRF_{C_nH_m} \times A_{C_nH_m}} \times 100\% \qquad \text{(S20)}$$

$$C \text{ balance} = \frac{C_{in} - C_{out}}{C_{in}} \times 100\% \qquad \text{(S21)}$$

Where $C_{CO_2,\text{ inlet}}$ and $C_{CO_2,\text{ outlet}}$ are the concentrations of CO_2 at the inlet and outlet, respectively. F_{inlet} and F_{outlet} are the inlet and outlet gas flow rates of the reactor. RRF is the relative response factor and A is the peak area of the species on chromatographic spectra.

Table S8: Catalytic performance of over different catalysts. Reaction conditions: 350 °C, 500 psig, 9000 $mlg_{cat}^{-1}h^{-1}$, H_2:CO_2 ratio 3:1, In_2O_3:HZSM-5 mass ratio 1:1.

Catalyst	CO selectivity (% C)	HC distribution (% mol C/ mol C)				CH_3OH selectivity (% C)	CO_2 conversion (% C)
		CH_4	C_2-C_4	$C_2^=$-$C_4^=$	C_{5+}		
**In_2O_3**	91.83	100.00	-	-	-	6.32	15.24
**HZSM-5**	-	-	-	-	-	-	-
**HZSM-5‖In_2O_3**	90.88	100.00	0.00	0.00	0.00	7.12	12.76
**In_2O_3‖HZSM-5**	91.24	33.61	32.62	5.41	28.35	1.53	20.79
**Micro_In_2O_3/HZSM-5**	77.11	4.41	37.66	6.63	51.30	4.86	19.88
**Nano_In_2O_3/HZSM-5**	84.71	100.00	0.00	0.00	0.00	11.45	12.00
**Micro_In_2O_3/0.3InZSM-5**	83.79	10.70	33.28	8.42	47.60	3.53	20.87
**Micro_In_2O_3/0.7InZSM-5**	84.00	14.22	33.82	8.87	43.10	4.29	19.45
**Micro_In_2O_3/3.5InZSM-5**	88.59	100.00	0.00	0.00	0.00	8.47	15.75
**Micro_In_2O_3/NaZSM-5**	92.52	100.00	0.00	0.00	0.00	5.67	16.60

Table S9: Catalytic performance of over different catalysts. Reaction conditions: 400 °C, 500 psig, 9000 $mlg_{cat}^{-1}h^{-1}$, H_2:CO_2 ratio 3:1, In_2O_3:HZSM-5 mass ratio 1:1.

**Catalyst**	**CO selectivity (% C)**	**HC distribution (% mol C/ mol C)**				**CH_3OH selectivity (% C)**	**CO_2 conversion (% C)**
		**CH_4**	**C_2-C_4**	**$C_2^=$-$C_4^=$**	**C_{5+}**		
**In_2O_3**	99.23	100.00	-	-	-	0.31	48.30
**HZSM-5**	-	-	-	-	-	-	-
**HZSM-5||In_2O_3**	98.82	100.00	0.00	0.00	0.00	0.74	45.00
**In_2O_3||HZSM-5**	98.35	66.78	23.64	6.23	3.35	0.33	30.03
**Micro_In_2O_3/HZSM-5**	85.23	12.14	48.93	9.69	29.25	3.66	32.76
**Nano_In_2O_3/HZSM-5**	97.60	100.00	0.00	0.00	0.00	0.69	40.08
**Micro_In_2O_3/0.3InZSM-5**	92.56	27.19	49.24	9.49	14.09	1.10	48.30
**Micro_In_2O_3/0.7InZSM-5**	92.81	33.13	41.21	9.32	16.34	1.40	44.21
**Micro_In_2O_3/3.5InZSM-5**	98.40	100.00	0.00	0.00	0.00	0.54	45.92
**Micro_In_2O_3/NaZSM-5**	98.93	100.00	0.00	0.00	0.00	0.49	48.63

Table S10: Catalytic performance of over different catalysts. Reaction conditions: 450 °C, 500 psig, 9000 $mlg_{cat}^{-1}h^{-1}$, H_2:CO_2 ratio 3:1, In_2O_3:HZSM-5 mass ratio 1:1.

**Catalyst**	**CO selectivity (% C)**	**HC distribution (% mol C/mol C)**				**CH_3OH selectivity (% C)**	**CO_2 conversion (% C)**
		**CH_4**	**C_2-C_4**	**$C_2^=$-$C_4^=$**	**C_{5+}**		
**In_2O_3**	99.45	100.00	-	-	-	0.05	51.33
**HZSM-5**	-	-	-	-	-	-	-
**HZSM-5‖In_2O_3**	99.50	100.00	0.00	0.00	0.00	0.11	50.00
**In_2O_3‖HZSM-5**	98.89	90.76	7.96	1.28	0.00	0.00	44.50
**Micro_In_2O_3/HZSM-5**	93.33	28.13	54.25	10.53	7.09	1.02	36.74
**Nano_In_2O_3/HZSM-5**	99.17	100.00	0.00	0.00	0.00	0.17	50.18
**Micro_In_2O_3/0.3InZSM-5**	98.55	69.01	27.79	3.20	0.00	0.06	47.72
**Micro_In_2O_3/0.7InZSM-5**	98.33	81.72	14.83	3.45	0.00	0.06	48.08
**Micro_In_2O_3/3.5InZSM-5**	99.04	100.00	0.00	0.00	0.00	0.13	46.07
**Micro_In_2O_3/NaZSM-5**	99.38	100.00	0.00	0.00	0.00	0.07	51.04

Table S11: Catalytic performance at different GHSV over micro_In_2O_3/HZSM-5. Reaction conditions: 350 °C, 500 psig, H_2:CO_2 ratio 3:1, In_2O_3:HZSM-5 mass ratio 1:1.

**GHSV ($mlg_{cat}^{-1}h^{-1}$)**	**Catalyst**	**CO selectivity (% C)**	**HC distribution (% mol C/mol C)**				**CH_3OH selectivity (% C)**	**CO_2 conversion (% C)**
			**CH_4**	**C_2-C_4**	**$C_2^=$-$C_4^=$**	**C_{5+}**		
4800	Micro_In_2O_3/HZSM-5	81.33	8.42	37.27	6.26	48.06	4.75	30.94
9000	Micro_In_2O_3/HZSM-5	77.11	4.41	37.66	6.63	51.30	4.86	19.88
12000	Micro_In_2O_3/HZSM-5	75.47	3.76	35.49	7.65	53.10	5.07	16.76

Table S12: Catalytic performance at different reaction pressure over micro_In_2O_3/HZSM-5. Reaction conditions: 350 °C, 9000 $mlg_{cat}^{-1}h^{-1}$, 500 psig, H_2:CO_2 ratio 3:1, In_2O_3:HZSM-5 mass ratio 1:1.

P (psig)	Catalyst	CO selectivity (% C)	HC distribution (% mol C/mol C)				CH_3OH selectivity (% C)	CO_2 conversion (% C)
			CH_4	C_2-C_4	$C_2^=$-$C_4^=$	C_{5+}		
500	Micro_In_2O_3/HZSM-5	83.76	4.56	36.65	6.51	52.28	3.20	29.13
450	Micro_In_2O_3/HZSM-5	87.04	3.73	38.09	7.65	50.53	2.44	26.53
300	Micro_In_2O_3/HZSM-5	93.60	3.28	43.95	15.96	36.81	1.03	24.36

Table S13: Catalytic performance at different H_2:CO_2 ratios over micro_In_2O_3/HZSM-5. Reaction conditions: 350 °C, 500 psig, In_2O_3:HZSM-5 mass ratio 1:1.

**H_2:CO_2**	**Catalyst**	**CO selectivity (% C)**	**HC distribution (% mol C/mol C)**				**CH_3OH selectivity (% C)**	**CO_2 conversion (% C)**
			**CH_4**	**C_2-C_4**	**$C_2^=$-$C_4^=$**	**C_{5+}**		
4:1	Micro_In_2O_3/HZSM-5	87.83	7.34	43.68	7.68	41.30	2.67	37.16
3:1	Micro_In_2O_3/HZSM-5	82.85	5.11	36.87	6.13	51.89	3.32	28.97
1:1	Micro_In_2O_3/HZSM-5	92.19	4.43	36.21	7.21	52.15	1.44	24.17

Table S14: Catalytic performance at different In_2O_3:HZSM-5 mass ratios over micro_In_2O_3/HZSM-5. Reaction conditions: 350 °C, 500 psig, H_2:CO_2 ratio 3:1.

In_2O_3:HZSM-5	Catalyst	CO selectivity (% C)	HC distribution (% mol C/mol C)				CH_3OH selectivity (% C)	CO_2 conversion (% C)
			CH_4	C_2-C_4	$C_2^=$-$C_4^=$	C_{5+}		
3:1	Micro_In_2O_3/HZSM-5	87.34	3.61	29.28	10.84	56.26	2.09	31.07
1:1	Micro_In_2O_3/HZSM-5	83.86	4.69	36.77	5.98	52.56	3.17	30.33
1:3	Micro_In_2O_3/HZSM-5	86.18	4.41	41.07	8.00	46.53	2.57	19.52

S7. C_{5+} HC distribution

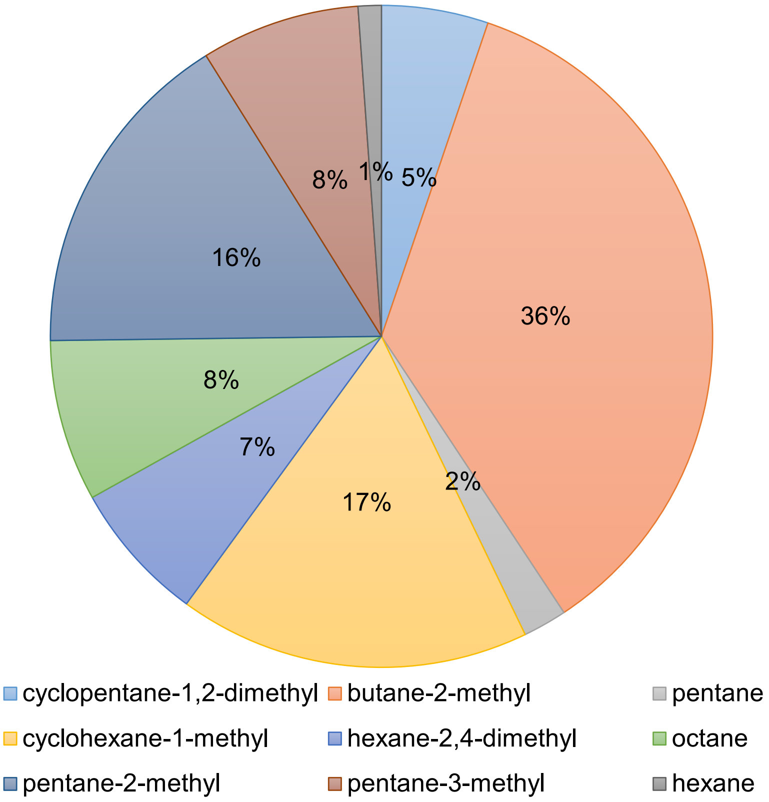

Figure S22: C_{5+} hydrocarbon distribution over micro_In_2O_3/HZSM-5. Reaction conditions: 350 ºC, 500 psig, 9000 $mlg_{cat}^{-1}h^{-1}$, H_2:CO_2 ratio 3:1, In_2O_3:HZSM-5 mass ratio 1:1.

Python code

Estimation of the distance between active sites in micro_In_2O_3/HZSM-5

```
# Distance estimation for micro_In2O3/HZSM-5
import numpy as np

# Distribution function of active sites
def F(r):
    return r**2.0 / (1.0 + np.exp(-r))

# Number of random points on the spherical catalyst particles
def random_points_on_sphere(num_points, radius):
    # Generate random angles
    theta = 2.0 * np.pi * np.random.rand(num_points)
    phi = np.arccos(2.0 * np.random.rand(num_points) - 1)

    # Calculate x, y, and z coordinates
    x = radius * np.sin(phi) * np.cos(theta)
    y = radius * np.sin(phi) * np.sin(theta)
    z = radius * np.cos(phi)

    return x, y, z

# Integration for total number of active sites
def compute_integral(r, dr):
    # Compute the integral of F(r) from r to r + dr using the trapezoidal rule
    integral = (F(r) + F(r + dr)) * dr / 2.0
    return integral


# Radius of shells
r1 = [50, 100, 200]

Res = []

for x in range(len(r1)):
    R1 = r1[x]
    num_points1 = F(R1)
    x1, y1, z1 = random_points_on_sphere(int(num_points1), R1)
    x1 += 200
    r2 = [50, 100, 200]
    for y in range(len(r2)):
        R2 = r2[y]
        print(R1, R2)
        num_points2 = F(R2)
        x2, y2, z2 = random_points_on_sphere(int(num_points2), R2)
        di_avg = 0
        for j in range(int(num_points2)):
            x_ref = x2[j]
            y_ref = y2[j]
            z_ref = z2[j]
```

```
        dist = 0
        for i in range(int(num_points1)):
            dist += np.sqrt((x1[i] - x_ref)**2 + (y1[i] - y_ref)**2 + (z1[i] - z_ref)**2)

        di_avg += dist / num_points1
    d = di_avg / num_points2
    Res.append([R1,R2,d])


for i in range(len(Res)):
    print(Res[i])

50 50
50 100
50 200
100 50
100 100
100 200
200 50
200 100
200 200
[50, 50, 207.8031410076237]
[50, 100, 220.7545877960515]
[50, 200, 271.26868903046966]
[100, 50, 220.5437869515818]
[100, 100, 232.22223862653422]
[100, 200, 282.2221649056947]
[200, 50, 271.5405001588666]
[200, 100, 282.3832852444298]
[200, 200, 325.65090051551726]
In [4]:
num = 0
den = 0
Vec = []
for elem in Res:
    num += (elem[2]*F(elem[0])*F(elem[1]))
    den += F(elem[0])*F(elem[1])
#    Vec.append([elem[2], F(elem[0])*F(elem[1])])

D = num/den

print(D)
#print(Vec)
303.57115380188765
In [ ]:
```

Python code

Estimation of the distance between active sites in nano_In_2O_3/HZSM-5

```
# Distance estimation for nano_In2O3/HZSM-5
import numpy as np


# Distribution function of active sites
def F(r):
    return r**2.0 / (1.0 + np.exp(-r))

# Number of random points on the spherical catalyst particles
def random_points_on_sphere(num_points, radius):
    # Generate random angles
    theta = 2.0 * np.pi * np.random.rand(num_points)
    phi = np.arccos(2.0 * np.random.rand(num_points) - 1)

    # Calculate x, y, and z coordinates
    x = radius * np.sin(phi) * np.cos(theta)
    y = radius * np.sin(phi) * np.sin(theta)
    z = radius * np.cos(phi)

    return x, y, z


# Integration for total number of active sites
def compute_integral(r, dr):
    # Compute the integral of F(r) from r to r + dr using the trapezoidal rule
    integral = (F(r) + F(r + dr)) * dr / 2.0
    return integral


# Radius of shells
r1 = [5, 10, 20]

Res = []

for x in range(len(r1)):
    R1 = r1[x]
    num_points1 = F(R1)
    x1, y1, z1 = random_points_on_sphere(int(num_points1), R1)

    x1 += 250

    r2 = [100, 200, 250]
    for y in range(len(r2)):
        R2 = r2[y]
        print(R1, R2)
        num_points2 = F(R2)
        x2, y2, z2 = random_points_on_sphere(int(num_points2), R2)
        di_avg = 0
```

```
    for j in range(int(num_points2)):
        x_ref = x2[j]
        y_ref = y2[j]
        z_ref = z2[j]
        dist = 0
        for i in range(int(num_points1)):
            dist += np.sqrt((x1[i] - x_ref)**2 + (y1[i] - y_ref)**2 + (z1[i] - z_ref)**2)

        di_avg += dist / num_points1
    d = di_avg / num_points2
    Res.append([R1,R2,d])


for i in range(len(Res)):
    print(Res[i])

5 100
5 200
5 250
10 100
10 200
10 250
20 100
20 200
20 250
[5, 100, 253.43283504666118]
[5, 200, 292.2553886546637]
[5, 250, 322.09993090699714]
[10, 100, 260.68041778419695]
[10, 200, 299.90942036481204]
[10, 250, 329.64720493184245]
[20, 100, 263.7132993121069]
[20, 200, 302.4046021358028]
[20, 250, 334.36108985655204]
In [3]:
num = 0
den = 0
Vec = []
for elem in Res:
    num += (elem[2]*F(elem[0])*F(elem[1]))
    den += F(elem[0])*F(elem[1])
#    Vec.append([elem[2], F(elem[0])*F(elem[1])])

D = num/den

print(D)
#print(Vec)
315.463330353002
```

References

(1) Brunauer, S.; Emmett, P. H.; Teller, E. Adsorption of Gases in Multimolecular Layers. *J. Am. Chem. Soc.* **1938**, *60* (2), 309-319. DOI: 10.1021/ja01269a023.
(2) Anton Paar QuantaTec, I. Instruction Manual and Safety Information. In *autosorb iQ Gas Sorption System*, Graz, Austria, 2022.
(3) Li, Y.; Wang, M.; Liu, S.; Wu, F.; Zhang, Q.; Zhang, S.; Cheng, K.; Wang, Y. Distance for communication between metal and acid sites for syngas conversion. *ACS Catalysis* **2022**, *12* (15), 8793-8801.
(4) Stöcker, M. Methanol-to-hydrocarbons: catalytic materials and their behavior. *Microporous and mesoporous materials* **1999**, *29* (1-2), 3-48.
(5) Bown, R. M.; Joyce, M.; Zhang, Q.; Reina, T. R.; Duyar, M. S. Identifying commercial opportunities for the reverse water gas shift reaction. *Energy Technology* **2021**, *9* (11), 2100554.
(6) Swapnesh, A.; Srivastava, V. C.; Mall, I. D. Comparative study on thermodynamic analysis of CO2 utilization reactions. *Chemical Engineering & Technology* **2014**, *37* (10), 1765-1777.
(7) Li, Z.; Qu, Y.; Wang, J.; Liu, H.; Li, M.; Miao, S.; Li, C. Highly selective conversion of carbon dioxide to aromatics over tandem catalysts. *Joule* **2019**, *3* (2), 570-583.
(8) Ghosh, S.; Sebastian, J.; Olsson, L.; Creaser, D. Experimental and kinetic modeling studies of methanol synthesis from CO2 hydrogenation using In2O3 catalyst. *Chemical Engineering Journal* **2021**, *416*, 129120.
(9) Song, W.; Nicholas, J. B.; Sassi, A.; Haw, J. F. Synthesis of the heptamethylbenzenium cation in zeolite-β: In situ NMR and theory. *Catal. Lett.* **2002**, *81* (1), 49-53.
(10) Park, J. W.; Lee, J. Y.; Kim, K. S.; Hong, S. B.; Seo, G. Effects of cage shape and size of 8-membered ring molecular sieves on their deactivation in methanol-to-olefin (MTO) reactions. *Applied Catalysis A: General* **2008**, *339* (1), 36-44.
(11) Arora, S. S.; Shi, Z.; Bhan, A. Mechanistic basis for effects of high-pressure H2 cofeeds on methanol-to-hydrocarbons catalysis over zeolites. *ACS Catalysis* **2019**, *9* (7), 6407-6414.
(12) Bird, R.; Stewart, W.; Lightfoot, E. Other mechanisms for mass transport. *Transport Phenomena, John Wiley & Sons, Inc* **2007**.
(13) Schechter, R. S. Transport Phenomena (Bird, R. Byron; Stewart, Warren E.; Lightfoot, Edwin N.). ACS Publications: 1961.
(14) Smith, J. M. Introduction to chemical engineering thermodynamics. ACS Publications: 1950.
(15) Kresse, G.; Furthmüller, J. Efficient iterative schemes for ab initio total-energy calculations using a plane-wave basis set. *Physical review B* **1996**, *54* (16), 11169.
(16) Perdew, J. P.; Burke, K.; Ernzerhof, M. Generalized gradient approximation made simple. *Physical review letters* **1996**, *77* (18), 3865.
(17) Kresse, G.; Joubert, D. From ultrasoft pseudopotentials to the projector augmented-wave method. *Physical review b* **1999**, *59* (3), 1758.
(18) Grimme, S.; Antony, J.; Ehrlich, S.; Krieg, H. A consistent and accurate ab initio parametrization of density functional dispersion correction (DFT-D) for the 94 elements H-Pu. *The Journal of chemical physics* **2010**, *132* (15).
(19) Kokotailo, G.; Lawton, S.; Olson, D.; Meier, W. Structure of synthetic zeolite ZSM-5. *Nature* **1978**, *272* (5652), 437-438.
(20) Gabrienko, A. A.; Arzumanov, S. S.; Moroz, I. B.; Prosvirin, I. P.; Toktarev, A. V.; Wang, W.; Stepanov, A. G. Methane activation on in-modified ZSM-5: the state of indium in the zeolite and pathways of methane transformation to surface species. *The Journal of Physical Chemistry C* **2014**, *118* (15), 8034-8043.

(21) Heyden, A.; Bell, A. T.; Keil, F. J. Efficient methods for finding transition states in chemical reactions: Comparison of improved dimer method and partitioned rational function optimization method. *The Journal of chemical physics* **2005**, *123* (22).
(22) Dang, S.; Qin, B.; Yang, Y.; Wang, H.; Cai, J.; Han, Y.; Li, S.; Gao, P.; Sun, Y. Rationally designed indium oxide catalysts for CO2 hydrogenation to methanol with high activity and selectivity. *Science advances* **2020**, *6* (25), eaaz2060.
(23) Murali, A.; Barve, A.; Leppert, V. J.; Risbud, S. H.; Kennedy, I. M.; Lee, H. W. Synthesis and characterization of indium oxide nanoparticles. *Nano Letters* **2001**, *1* (6), 287-289.
(24) Al-Dughaither, A. S.; de Lasa, H. HZSM-5 zeolites with different SiO2/Al2O3 ratios. Characterization and NH3 desorption kinetics. *Industrial & Engineering Chemistry Research* **2014**, *53* (40), 15303-15316.
(25) Wang, B.; Wang, H.; Liu, G.; Li, X.; Wu, J. Conversion of dimethyl ether to toluene under an O 2 stream over W/HZSM-5 catalysts. *Catalysis Science & Technology* **2015**, *5* (3), 1813-1820.
(26) Jawad, A.; Ahmed, S. Analysis and process evaluation of metal dopant (Zr, Cr)-promoted Ga-modified ZSM-5 for the oxidative dehydrogenation of propane in the presence and absence of CO 2. *RSC advances* **2023**, *13* (16), 11081-11095.
(27) Kelgenbaeva, Z.; Khandaker, J. I.; Ihara, H.; Omurzak, E.; Sulaimankulova, S.; Mashimo, T. Thermal and optical properties of In and In2O3 nanoparticles synthesized using pulsed plasma in water. *physica status solidi (a)* **2018**, *215* (11), 1700910.
(28) Kar, S.; Santra, S.; Chaudhuri, S. Direct synthesis of indium nanotubes from indium metal source. *Crystal Growth and Design* **2008**, *8* (1), 344-346.
(29) Avivi, S.; Mastai, Y.; Gedanken, A. Sonohydrolysis of In3+ ions: formation of needlelike particles of indium hydroxide. *Chemistry of materials* **2000**, *12* (5), 1229-1233.
(30) Xie, T.; Ding, J.; Shang, X.; Zhang, X.; Zhong, Q. Effective synergies in indium oxide loaded with zirconia mixed with silicoaluminophosphate molecular sieve number 34 catalysts for carbon dioxide hydrogenation to lower olefins. *Journal of Colloid and Interface Science* **2023**, *635*, 148-158.
(31) Zhou, X.; Xu, Z.; Zhang, T.; Lin, L. The chemical status of indium in indium impregnated HZSM-5 catalysts for the SCR of NO with CH4. *Journal of Molecular Catalysis A: Chemical* **1997**, *122* (2-3), 125-129.
(32) Wang, Y.; Wang, G.; van der Wal, L. I.; Cheng, K.; Zhang, Q.; de Jong, K. P.; Wang, Y. Visualizing Element Migration over Bifunctional Metal-Zeolite Catalysts and its Impact on Catalysis. *Angewandte Chemie* **2021**, *133* (32), 17876-17884.
(33) Shen, C.; Sun, K.; Zou, R.; Wu, Q.; Mei, D.; Liu, C.-j. CO2 Hydrogenation to Methanol on Indium Oxide-Supported Rhenium Catalysts: The Effects of Size. *ACS Catalysis* **2022**, *12* (20), 12658-12669.
(34) Kaur, M.; Jain, N.; Sharma, K.; Bhattacharya, S.; Roy, M.; Tyagi, A.; Gupta, S.; Yakhmi, J. Room-temperature H2S gas sensing at ppb level by single crystal In2O3 whiskers. *Sensors and Actuators B: Chemical* **2008**, *133* (2), 456-461.
(35) Bielz, T.; Lorenz, H.; Jochum, W.; Kaindl, R.; Klauser, F.; Klotzer, B.; Penner, S. Hydrogen on In2O3: reducibility, bonding, defect formation, and reactivity. *The Journal of Physical Chemistry C* **2010**, *114* (19), 9022-9029.
(36) Wang, W.; Qu, Z.; Song, L.; Fu, Q. An investigation of Zr/Ce ratio influencing the catalytic performance of CuO/Ce1-xZrxO2 catalyst for CO2 hydrogenation to CH3OH. *Journal of Energy Chemistry* **2020**, *47*, 18-28.
